# Human embryo implantation

**DOI:** 10.1242/dev.201507

**Published:** 2023-05-31

**Authors:** Joanne Muter, Vincent J. Lynch, Rajiv C. McCoy, Jan J. Brosens

**Affiliations:** ^1^ Division of Biomedical Sciences, Warwick Medical School, University of Warwick, Coventry, CV2 2DX, UK; ^2^ Tommy's National Centre for Miscarriage Research, University Hospitals Coventry & Warwickshire NHS Trust, Warwick Medical School, University of Warwick, Coventry, CV2 2DX, UK; ^3^ Department of Biological Sciences, University at Buffalo, Buffalo, NY 14260-4610, USA; ^4^ Department of Biology, Johns Hopkins University, Baltimore, MD 21218, USA

**Keywords:** Aneuploidy, Decidualisation, Implantation, Pregnancy, Uterine remodelling

## Abstract

Embryo implantation in humans is interstitial, meaning the entire conceptus embeds in the endometrium before the placental trophoblast invades beyond the uterine mucosa into the underlying inner myometrium. Once implanted, embryo survival pivots on the transformation of the endometrium into an anti-inflammatory placental bed, termed decidua, under homeostatic control of uterine natural killer cells. Here, we examine the evolutionary context of embryo implantation and elaborate on uterine remodelling before and after conception in humans. We also discuss the interactions between the embryo and the decidualising endometrium that regulate interstitial implantation and determine embryo fitness. Together, this Review highlights the precarious but adaptable nature of the implantation process.

## Teaser

**Summary:** This Review examines the foundational processes of pregnancy from an evolutionary perspective, highlights uterine remodelling permissive for implantation, and examines the impact of embryo fitness on endometrial homeostasis.

## Introduction

**Fig. 1.**
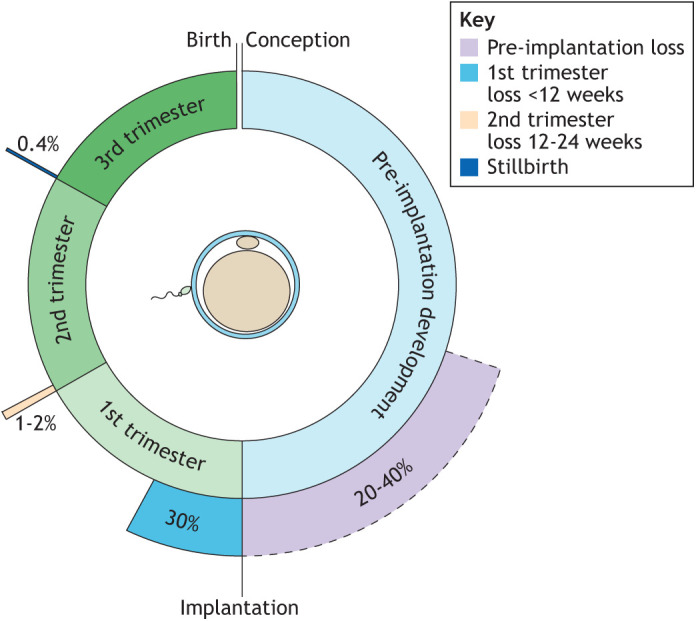
**Reproductive failure and success.** Estimates of the rates of pre-implantation embryo loss, miscarriage before and after 12 weeks of gestation, and stillbirth.

Human reproduction has been described as disappointingly inefficient (Evers, 2002; Macklon et al., 2002). This statement appears justifiable as only 40-60% of all conceptions survive to birth in young healthy women (Hertig and Bernischke, 1967; Zinaman et al., 1996; Norwitz et al., 2001; Jarvis, 2016). Once the embryo embeds in the endometrium, human chorionic gonadotrophin (hCG) rises in maternal blood and urine, thus allowing for robust estimates of the incidence of pregnancy loss. There is remarkable agreement among many studies that one in three embryos perish after implantation (Wilcox et al., 1999; Wang et al., 2003; Foo et al., 2020; Zinaman et al., 1996). More than half of pregnancy losses occur so early that they escape detection with little or no discernible impact on maternal reproductive fitness beyond increasing the likelihood of conception in subsequent cycles (Wang et al., 2003). The remaining losses present as clinical miscarriages, 90-95% of which occur in the first 12 weeks of pregnancy (Fig. 1) (Magnus et al., 2019). Estimates of the rates of embryo attrition prior to implantation are challenging and dependent on variables that determine the likelihood of fertilisation, including the frequency of intercourse during the fertile window of the menstrual cycle (Dunson et al., 2002; Jarvis, 2016). Nevertheless, with conception rates per cycle of 40% or less in young women who are trying to conceive, ‘normal’ rates of pre-implantation embryo loss between 20 and 40% appear reasonable estimates (Fig. 1).

There is no evidence that the incidence of early pregnancy loss, when stratified by maternal age, has changed in recent decades, or varies significantly by geography (Hardy et al., 2016). Maternal age, however, has an outsized impact on the likelihood of a successful pregnancy, which is accounted for by the impacts of meiotic errors in oocytes on pre- and post-implantation embryo loss (reviewed by Brosens et al., 2022). In addition to maternal age, prior adverse reproductive events are also associated with increased risk of pregnancy failure. For example, the risk of miscarriage increases stepwise by 7-9% with each previous pregnancy loss independently of maternal age or other variables (Kolte et al., 2021; Magnus et al., 2019). It is important to note that, even after five consecutive clinical miscarriages before the age of 34 years, the likelihood of live birth in the next pregnancy still exceeds 50% (Kolte et al., 2021). In *in vitro* fertilisation (IVF), the likelihood of pregnancy also declines in function of the number of previous implantation failures, although the effect sizes are modest when compared with the impact of miscarriages (Smith et al., 2015).

Thus, human reproduction is defined by high failure rates but also good cumulative success rates, reflecting a physiological system adapted to select against low-fitness offspring (Brosens et al., 2022). All mammals employ strategies to mitigate against the risk of investment in unfit offspring (Box 1). Intrinsic chromosomal instability in human embryos (Box 2) may have necessitated the emergence of a highly responsive and malleable uterine environment, underpinned by a suite of evolved traits, including cyclical menstruation, spontaneous decidualisation, and embryo biosensing and selection (Brosens et al., 2022). Here, we examine the evolutionary framework of these human reproductive traits and discuss how emerging insights into the cellular dynamics and fate decisions in the endometrium are fundamentally altering our understanding of implantation.

**Box 1. Physiological versus pathological pregnancy failure**

Pregnancy is a costly endeavour in all mammals. For example, maternal energy expenditure in human pregnancy is close to physiological limits and comparable to that of endurance athletes (Thurber et al., 2019). Not surprisingly, mechanisms to temporarily suppress reproduction evolved in all mammals to protect the mother against prolonged investment in a failing pregnancy or unfit offspring (Wasser and Barash, 1983). In humans, reproductive suppression can involve inhibition of the hypothalamic-pituitary-ovarian axis, especially in adolescent women (resulting in delayed menarche or anovulation) (Beehner and Lu, 2013) and elimination of the conceptus at implantation (embryo selection) (Macklon and Brosens, 2014) or in early pregnancy (e.g. aneuploid miscarriage) (Brosens et al., 2022). Other species developed alternative strategies, such as delayed fertilisation (sperm storage) (Wimsatt, 1975) and delayed embryo implantation (embryonic diapause) (Renfree and Fenelon, 2017). Importantly, reproductive suppression relies on physiological mechanisms that sense deleterious (internal/external) cues, which is fundamentally different from reproductive failure caused by disease or trauma. As articulated by David Haig, natural selection acting on mothers favours physiological processes that increase the overall number of surviving offspring, not necessarily the survival of an individual offspring (Haig, 1993; Haig, 2019). In humans, physiological embryo selection is easily conflated with pathology. For example, repeated implantation failure of low-fitness IVF embryos can lead to the diagnosis of ‘recurrent implantation failure’, an ill-defined clinical label that often spurs uninformative investigations and ineffective ad hoc treatments (Polanski et al., 2014; Coughlan et al., 2014). Conversely, pathological relaxation of embryo selection at implantation has been linked to recurrent miscarriage and associated obstetrical disorders, such as preterm birth (Brosens et al., 2022; Bortoletto et al., 2022).

**Box 2. Chromosome instability in pre-implantation human embryos**

Aneuploidy, the gain or loss of entire chromosomes compared with the typical 46-chromosome complement, is the primary cause of embryonic loss and fetal demise. The impact of aneuploidy on embryonic fitness depends on the origins and type of errors, and the fate of abnormal blastomeres. The incidence of human aneuploidies originating during the formation of the haploid egg (meiosis) is strongly associated with maternal age (Webster and Schuh, 2017). By contrast, aneuploidy of paternal meiotic origin is relatively rare and paternal age independent (McCoy et al., 2015). Numerous aneuploidies also arise during post-zygotic cell divisions, generating ‘mosaic’ embryos possessing both normal and aneuploid cells. Aneuploidy alters the dosage of genes on the affected chromosome and impacts on downstream regulatory cascades. Thus, the fitness impacts of aneuploidy (or any other mutation) are highly context dependent (Li and Zhu, 2022). During human embryonic development, aneuploidy drives natural selection at the level of both embryos and cells. Rates of both meiotic- and mitotic-origin aneuploidy decline through development owing, in part, to embryonic mortality (McCoy et al., 2022). Recent studies demonstrated that, although meiotic aneuploidies and severe mitotic aneuploidies are often embryonically lethal (Gruhn et al., 2019; McCoy et al., 2015), low levels of mosaicism are common in normal development (Greco et al., 2015; Viotti et al., 2021). Within mosaic embryos, selection operates against aneuploid cell lineages as they are progressively diluted by euploid cells. This model is supported by evidence of chromosomal mosaicism in mice (Bolton et al., 2016; Singla et al., 2020), human embryos and gastruloids at peri- and post-implantation stages of development (Shahbazi et al., 2020; Popovic et al., 2019; Starostik et al., 2020; Yang et al., 2021). The intensity and nature of selection processes appear to be aneuploidy and cell-type specific (Shahbazi et al., 2020). For a more detailed discussion, see Brosens et al. (2022).

## Evolution of embryo implantation

Over 170 million years or more of eutherian (placental mammal) evolution, a spectacular array of reproductive strategies has emerged, resulting in species-specific differences in pre-implantation embryo development, placental structures, litter size, gestational length, as well as mechanisms that govern implantation and parturition. Rapid reproductive innovation in placental mammals is attributed to co-option of species-specific transposable elements (TEs) into the regulatory DNA landscape of pre-implantation embryos, extra-embryonic tissues and endometrial cells (Senft and Macfarlan, 2021), leading to gain and loss of numerous reproductive genes (Lynch et al., 2011; Mika et al., 2021). In this section, we summarise the evolutionary origins of implantation, examine the foundational processes that enabled the formation of a stable maternal-fetal interface and elaborate on how evolved traits, such as spontaneous decidualisation and menstruation, shaped the implantation process in humans.

### Implantation evolved from inflammation

**Fig. 2.**
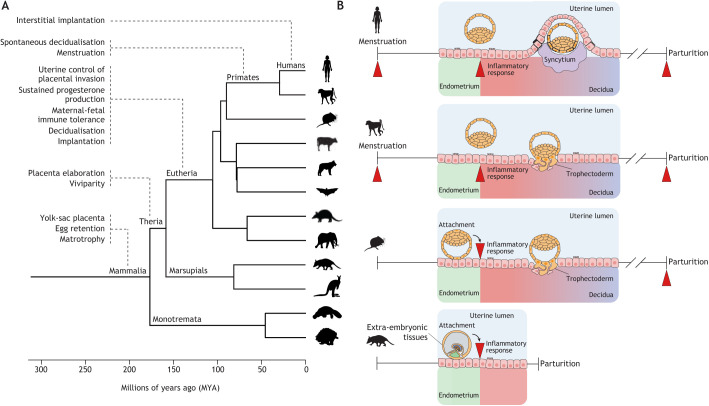
**Evolution of embryo implantation.** (A) Phylogenetic tree showing major reproductive innovations in the evolution of pregnancy in Mammalia, Theria, Eutheria and higher primates, including humans (adapted from Mika et al., 2022). (B) Attachment of extra-embryonic tissues to the endometrial luminal epithelium triggers uterine inflammation (red arrowhead) and parturition in marsupials, such as the grey short-tailed opossum (bottom). In eutherians with haemochorial placenta, such as mice, inflammatory reprogramming of endometrial stromal cells upon embryo attachment gives rise to decidual cells that accommodate invasive trophectoderm (placental precursors) throughout gestation. Consequently, pregnancy in eutherians is demarcated by two inflammatory uterine events, coinciding with implantation and parturition (note the two arrowheads). The emergence of an endogenous (endometrial) deciduogenic signal leads to spontaneous decidualisation and accounts for menstruation in higher primates. Menstruation imposes an additional inflammatory event on the uterus that marks the start of each reproductive (menstrual) cycle (note the three arrowheads). In humans and great apes (top), endometrial invasion is not limited to placental cells, but the entire embryo embeds in the stroma at implantation (interstitial implantation).

Mammalian pregnancy evolved in the therian stem lineage, before the common ancestor of marsupials and eutherian mammals (Fig. 2A) (Chavan et al., 2021). In marsupials, such as the grey short-tailed opossum (*Monodelphis domestica*), the duration of pregnancy is shorter than the reproductive cycle, and steroid hormone profiles before and during pregnancy are indistinguishable. For much of the pregnancy, the marsupial embryo develops within a shell coat, which hatches towards the end of gestation. Subsequent attachment of the extra-embryonic membranes to the endometrial luminal epithelium triggers an inflammatory uterine response that leads to parturition and birth of altricial young (Fig. 2B) (Hansen et al., 2016; Griffith et al., 2017; Stadtmauer and Wagner, 2020a). By contrast, pregnancy in humans and other eutherians is demarcated by two inflammatory uterine events, corresponding to embryo implantation and parturition, respectively. Pregnancy is further characterised by a unique steroid hormone profile and the suspension of the reproductive cycle, which resumes following parturition. Thus, inflammation caused by attachment of extra-embryonic cells to the endometrial luminal epithelium serves as the ancestral signal for uterine recognition of pregnancy in both eutherian and marsupial mammals. In marsupials, this pro-inflammatory response is unrestrained and leads to parturition, whereas suppression of mucosal inflammation following embryo attachment and implantation in eutherians enabled the formation of a stable uterine–placental interface, the extension of pregnancy beyond the reproductive cycle and the birth of precocial offspring (Griffith et al., 2017; Chavan et al., 2021).

### Suppression of uterine inflammation

Domestication of uterine inflammation upon embryo attachment and invasive implantation required a suite of maternal adaptations, including prolonged ovarian progesterone production, uterine control of placental invasion, immune tolerance and decidual transformation of the endometrium (Fig. 2A). We next discuss the broad mechanisms and evolution of these core processes in the context of human pregnancy.

#### Progesterone production

Progesterone is produced by the corpus luteum, which forms from the empty follicle following ovulation and regresses at the end of the reproductive cycle. Notably, the transition from oviparity to viviparity was facilitated by the evolution of progesterone production by the corpus luteum to promote retention of the conceptus in the reproductive tract (Rothchild, 2003). All eutherians depend on sustained progesterone signalling to suppress endometrial inflammation and maintain uterine quiescence throughout pregnancy. This is achieved either by extending the life span of the ovarian corpus luteum, outsourcing progesterone production to another organ such as the placenta, or both. For example, in humans, the implanting embryo secretes an abundance of hCG, which temporarily rescues ovarian progesterone production until the placenta takes over at around 6-8 weeks of pregnancy, a process known as the luteo-placental shift (Csapo et al., 1973). CGB genes, encoding the biologically active β-subunit of hCG, first arose in the common ancestor of the higher primates as the result of duplication of *LHB* (encoding luteinising hormone, LH). Humans have six transcriptionally active CBG genes encoding different paralogues, the highest number among primates (Casarini et al., 2018). By contrast, the corpus luteum in mice remains the only source of progesterone in pregnancy (Mesiano, 2022).

Steroid hormones, including oestradiol and progesterone, act by binding their cognate nuclear receptors, members of steroid/thyroid hormone superfamily of ligand-dependent transcription factors (Brosens et al., 2004). Loss of progesterone receptor activity in the pregnant uterus is the universal parturition signal in eutherians. In mice, this is achieved by involution (luteolysis) of the corpus luteum, resulting in a precipitous drop in circulating progesterone levels. In humans, however, progesterone levels are maintained until after the delivery and the onset of labour likely reflects loss of progesterone receptor activity in response to inflammatory uterine stress signals (Mesiano, 2022). Notably, treatment of pregnant mice with exogenous progesterone only delays the onset of labour as, akin to humans, activation of uterine-intrinsic inflammatory pathways renders parturition inevitable (Siewiera et al., 2023).

#### Uterine control of placental invasion

In eutherians, the maternal–fetal interface is formed by specialised placental epithelial cell lineages, termed trophoblast, that access and modify uterine structures to ensure gas exchange, flow of substrates to the fetus and disposal of waste products (Soares et al., 2018). Phylogenetic reconstructions intimated that the first eutherians had invasive placentas with a haemochorial interface (Wildman et al., 2006; Elliot and Crespi, 2006; Mika et al., 2022), meaning that trophoblast cells penetrate the endometrial stroma and its vasculature to access maternal blood. The ancestral haemochorial interface is maintained in rodents, humans and other higher primates, whereas other species evolved less invasive (endotheliochorial) or non-invasive (epitheliochorial) placentas in which the trophoblast interfaces with endometrial endothelial cells (e.g. cats, dogs and other carnivores) or luminal epithelial cells (e.g. lower primates, such as lemurs and lorises), respectively (Carter and Enders, 2004). In mice and most primates, trophectoderm cells (precursors of placental lineages) breach the luminal epithelium and invade the underlying endometrial stroma to various degrees, but the developing embryo remains in the uterine cavity (Fig. 2B). Humans and great apes, however, exhibit interstitial implantation and deep placentation, meaning that the entire embryo embeds in the endometrial stroma and invading (extravillous) trophoblast cells penetrate beyond the endometrium into the inner third of the myometrium, also known as the uterine junctional zone (reviewed by Carter et al., 2015). Extravillous trophoblast invasion, which is both interstitial and endovascular, crucially ensures that maternal blood supply of the human placenta keeps pace with the requirements of the growing fetus by converting the decidual and junctional zone portions of the uterine spiral arteries into high-capacity fibrinoid conduits (Brosens et al., 2002). Remarkably, endometrial gene expression patterns in both living and reconstructed ancestral mammals correlate with the degree of placental invasiveness, indicating that, rather than acting as a passive substrate, the uterus controls implantation and the invasiveness of trophoblast (Stadtmauer and Wagner, 2020b; Mika et al., 2022). Further, emerging evidence suggests that the differences in endometrial resistance to trophoblast invasion between placental mammals correlate with the prevalence in different species of metastatic cancer, a process also characterised by stromal invasion of (malignant) epithelial cells (Kshitiz et al., 2019; Suhail et al., 2022).

#### Immune tolerance

The embryo and placenta express a combination of maternal and paternal antigens; this combination is referred to in immunological terms as semi-allogeneic. The immune system of eutherian mammals, therefore, had to evolve mechanisms to prevent immune-mediated rejection of the semi-allogeneic conceptus (Gobert and Lafaille, 2012). Co-option of novel TEs to rewire gene networks contributed to resolution of this so-called ‘immunological paradox of pregnancy’ (Male, 2021). For example, insertion of CNS1, a eutherian-specific TE, in the *FoxP3* locus enabled the differentiation of naïve CD4^+^ T cells into regulatory T (Treg) cells at the maternal–fetal interface (Samstein et al., 2012). Although Treg cells express antigen-specific T-cell receptors, their activation dampens local immune responses through bystander suppression (Kahn and Baltimore, 2010). Emergence of placental mammals also coincided with the innovation of a high-affinity ligand (PD-L2; PDCD1LG2) for programmed cell death protein (PD-1; PDCD1), an inhibitory T-cell receptor and crucial modulator of adaptive immunity (Philips et al., 2020). In humans, multiple additional mechanisms shore up tolerance to placental alloantigens, including T-cell inactivation through indoleamine 2,3-dioxygenases (IDO)-dependent tryptophan deprivation, secretion of immunosuppressive mediators [such as glycodelin A (PAEP), prostaglandin E2 (PGE2), TGFβ and galectin 1], entrapment of antigen-presenting immune cells at the maternal–fetal interface, and the absence of HLA class I and II allotypes on non-invading trophoblasts (reviewed by Moffett and Shreeve, 2022). Further, in both humans and mice, trophoblast antigens are decorated with immunosuppressive glycans, which suppress a systematic immune response when shed into the maternal circulation (Rizzuto et al., 2022)

#### Decidualisation

Haemochorial placentation coincided with the inflammatory reprogramming of endometrial stromal cells into progesterone-dependent decidual cells (Lynch et al., 2015; Erkenbrack et al., 2018; Chavan et al., 2021), a process that involves massive changes in gene expression under the control of novel TE-derived regulatory elements that confer responsiveness to steroid hormones (Mika and Lynch, 2022). Decidual cells are defined by their epithelioid morphology, resistance to oxidative and metabolic stress signals and secretory phenotype (Gellersen and Brosens, 2014; Kajihara et al., 2014). They form the decidua in pregnancy, which serves as the maternal bed for the invading placenta. The decidua can be shallow or deep, often reflecting the depth of trophoblast invasion in different species (Ramsey et al., 1976), and, like the placenta, it is cast off at parturition (Gellersen and Brosens, 2014).

Decidual transformation of the endometrium starts with an inflammatory tissue reaction, which synchronises the differentiation of stromal cells with the recruitment of bone marrow-derived mesenchymal stem/progenitor cells (BM-MSCs) (Gellersen and Brosens, 2014; Tal et al., 2019; Diniz-da-Costa et al., 2021) and natural killer (NK) cells (Strunz et al., 2021). NK cells are effector lymphocytes of the innate immune system involved in controlling microbial infections, elimination of stressed and malignant cells, and allorecognition (Vivier et al., 2008). In the endometrium, decidualising stromal cells regulate the proliferative expansion and differentiation of NK cells into functionally and phenotypically distinct uterine NK (uNK) subsets that are intrinsically tolerant to invading trophoblast cells and secrete an abundance of cytokines and angiogenic factors implicated in vascular remodelling (Strunz et al., 2021; Huhn et al., 2020; Moffett and Shreeve, 2022). Remarkably, uNK cells can kill intracellular bacteria in placental trophoblast without compromising the viability of host cells (Crespo et al., 2020). Thus, the emergence of decidual cells in ancestral eutherians not only enabled invasive implantation but converted allorecognition of trophoblast cells by uNK cells into a process that facilitates haemochorial placentation and safeguards the maternal–fetal interface against infection.

#### Spontaneous decidualisation and menstruation

**Fig. 3.**
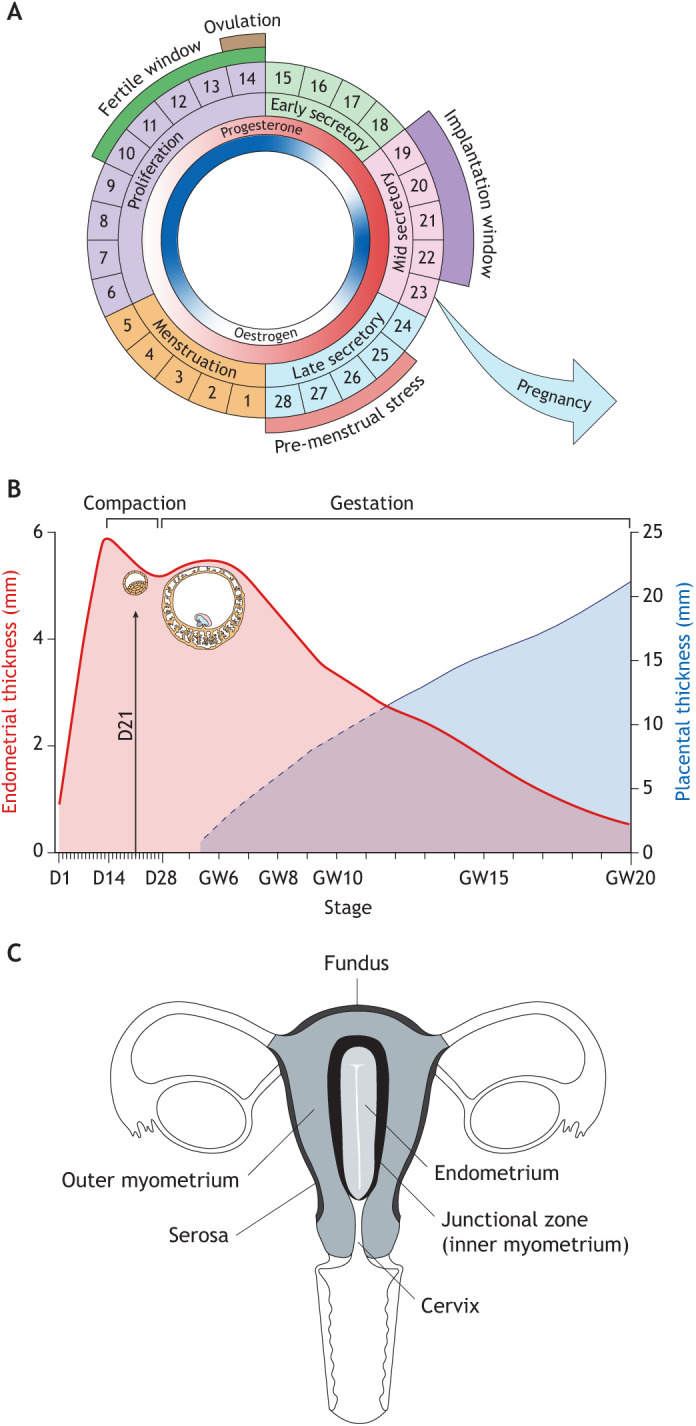
**Uterine remodelling.** (A) The menstrual cycle. The cycle is depicted as being 28 days long, although variations in cycle length are common between women and across the reproductive years (Bull et al., 2019). The rise and fall in oestradiol and progesterone levels are indicated in the inner circles. The fertile window indicates the days preceding ovulation when intercourse is most likely to result in pregnancy. The window of implantation coincides with the mid-secretory phase of the cycle. (B) Dynamic changes in endometrial thickness from menstruation until 20 weeks of pregnancy. Superimposed are the diameters of the conceptus at implantation and 6 weeks of pregnancy, as well as placental thickness. This composite figure is based on data extracted from several studies (Raine-Fenning et al., 2004a; Tongsong and Boonyanurak, 2004). D, day of menstrual cycle; GW, gestational week. (C) Uterine zonal anatomy based on T2-weighted magnetic resonance imaging.

Decidualisation in most species is triggered by attachment of the blastocyst to the luminal endometrial epithelium (Wang and Dey, 2006). A conspicuous innovation in Catarrhine primates (humans, apes and Old-World monkeys) is ‘spontaneous’ decidualisation, meaning that the decidual reaction is initiated in each reproductive cycle irrespective of embryo attachment (Fig. 2A) (Emera et al., 2012; Catalini and Fedder, 2020). An inevitable consequence of spontaneous decidualisation, which is restricted to the upper endometrial layer, is menstruation (Fig. 2B), defined as bleeding caused by partial tissue shedding in response to falling progesterone levels in non-conception cycles. Outside the primate lineage, menstruation evolved in a handful of other species, including a small number of bats, the elephant shrew (*Elephantulus myurus*) and the spiny mouse (*Acomys cahirinus*), the only known menstruating rodent out of 2277 species (Critchley et al., 2020). Menstruation co-evolved with other reproductive traits, including extended copulation and spontaneous ovulation (Emera et al., 2012; Catalini and Fedder, 2020). The onset of bleeding marks the start of the menstrual cycle (Fig. 3A). Menstruation is followed by a proliferative or follicular phase, during which ovarian oestradiol production synchronises the regeneration of the superficial endometrial layer with the timing of the LH surge and ovulation (Shoham et al., 1995). Ovulation in menstruating primates is characterised by a steep rise in circulating progesterone levels (Bellofiore et al., 2018), which heralds the start of the secretory or luteal phase of the cycle.

The purpose of cyclical menstruation has been debated over many years, with an emerging consensus coalescing around the idea it is a non-adaptive consequence of spontaneous decidualisation, meaning that menstruation serves no particular purpose beyond enabling initiation of a new cycle (Finn, 1998; Emera et al., 2012). However, menstruation is an inflammatory process that results in rapid tissue turnover and cyclical rejuvenation involving activation of tissue-resident mesenchymal and epithelial progenitor cells that reside in the basal layer (Cousins et al., 2022). It is now well established that inflammation etches lasting marks within tissues by increasing stemness and bolstering future inflammatory responses through epigenetic mechanisms (Naik and Fuchs, 2022). At the tissue level, this inflammatory ‘memory’ may explain the phenomenon of pre-conditioning or hormesis, which refers to the observation that repeated exposures of any organ to low levels of stress confers resistance to stress levels that otherwise cause tissue damage (Brosens et al., 2009). Indirect evidence that menstruation ‘preconditions’ the uterus for pregnancy emerged from studies in adolescent women, demonstrating an inverse correlation between the incidence of pregnancy disorders and gynecological age (the interval between the menarche and first pregnancy) (Brosens et al., 2017). Menstruation imposes an important novel hurdle on the implantation process: the wholesale transformation of a mucosa programmed for cyclical breakdown into a decidua capable of accommodating an invasive placenta throughout gestation (Brosens et al., 2022).

## Embryo implantation

Tracing the evolution of embryo implantation highlighted the dependency of human pregnancy on intense uterine remodelling before and after conception. Here, we discuss the gross changes in uterine structures that precede embryo implantation and elaborate how endometrial regeneration prior to ovulation establishes a spatial template for interstitial embryo implantation and deep placentation. We then focus on how progesterone-dependent differentiation of the endometrium creates a pro-inflammatory implantation window, after which the endometrium either breaks down or becomes transformed into the decidua of pregnancy (Fig. 3A). We examine how decidualising stromal cells control the fate of the endometrium upon closure of the implantation window before clarifying how the processes involved in interstitial embryo implantation also safeguard the mother against investment into low-fitness embryos (Box 1).

### Gross uterine remodelling

In each ovulatory cycle, oestradiol-dependent proliferation of the endometrium followed by progesterone-dependent differentiation culminates in a short window during which embryo implantation can take place. In humans, the implantation window opens 6 days after the pre-ovulatory LH surge and lasts for approximately 4 days, which corresponds to days 19-22 of a standardised 28-day cycle (Fig. 3A). Following rapid oestrogen-dependent growth during the proliferative phase, the post-ovulatory rise in progesterone levels leads to discrete compaction of the endometrium prior to the implantation window (Fig. 3B). Upon embryo implantation, the decidua accommodating the invading placenta, termed ‘decidua basalis’, compacts further into a thin and increasingly stiff layer (Fig. 3B). By contrast, the stiffness (elastic modulus) of the decidua away from the placenta (decidua parietalis) is a magnitude lower compared with the decidua basalis and is comparable to non-pregnant endometrium (Abbas et al., 2019).

Hormone-dependent tissue remodelling before and after implantation also encompasses the junctional zone myometrium (Brosens et al., 2002), a specialised layer of circular smooth muscle that surrounds the endometrium (Weiss et al., 2006). Unlike the outer myometrium, the junctional zone is derived from the same embryological mesenchymal precursor as endometrial stromal cells (Konishi et al., 1984). During reproductive years, the junctional zone can be visualised by high-resolution ultrasound or T2-weighted magnetic resonance imaging (MRI) (Fig. 3C). The thickness of the junctional zone increases in response to oestrogen signalling, albeit less pronounced compared with the endometrium (Brosens et al., 1998). It generates anatomically confined peristaltic contraction waves in a cycle-dependent manner (Brosens et al., 1995). In the proliferative phase, junctional zone contractions start predominantly near the cervix and are propagated towards the uterine fundus (cervico-fundal). Increased amplitude of these waves during the fertile window facilitates sperm transport towards the Fallopian tube on the side of the pre-ovulatory follicle (Kunz et al., 1996; Kunz et al., 1998). Following ovulation, short asymmetrical contraction waves may play a role in ensuring that implantation occurs near the fundus of the uterine cavity, whereas fundo-cervical peristalsis prior and during menstruation likely controls the flow of effluent (Brosens et al., 1998). The junctional zone changes dramatically in pregnancy. Focal disruption of this smooth muscle layer has been observed by MRI around the time of embryo implantation, well in advance of trophoblast invasion of the myometrium (Turnbull et al., 1995). In pregnancy, increased signal intensity renders the junctional zone indistinct on imaging. Electron microscopy studies have demonstrated that myofibroblasts are present at the endometrial–myometrial junction. These cells acquire characteristics of smooth muscle cells in the luteal phase and in early pregnancy, suggesting that the junctional zone remodelling involves fibroblast-to-smooth muscle trans-differentiation (Konishi et al., 1984; Fujii et al., 1989; Brosens et al., 2002). In pregnancy, a subset of stromal cells in the decidua spongiosa adjacent to myometrium abundantly express canonical smooth muscle genes, including *ACTA2* (smooth muscle alpha-actin), *CNN1* (calponin 1) and *MYLK* (myosin light chain kinase) (Vento-Tormo et al., 2018), raising the possibility that the appearance of myometrial trophoblast invasion reflects, at least in part, upwards trans-differentiation of stromal cells into myocytes. Histologically, disruption of the junctional zone in pregnancy starts in the centre of the placental bed, which already harbours an abundance of cytotrophoblast and immune cells by the eighth week of pregnancy, and spreads laterally like ‘ripples created by a stone dropped into a still pool of water’ (Pijnenborg et al., 1981).

Thus, gross structural changes in uterine zonal anatomy precede and follow embryo implantation. Insufficient endometrial growth prior to ovulation, absence of endometrial compaction following ovulation, and lack of peri-implantation junctional zone remodelling are all associated clinically with increased risk of implantation failure (Zilberberg et al., 2020; Craciunas et al., 2019; Lesny et al., 1999). Next, we discuss how endometrial regeneration during the menstrual and proliferative phase establishes a spatial template in preparation for interstitial embryo implantation and deep placentation.

### Endometrial regeneration

#### Menstruation

Menstruation (reviewed by Salamonsen et al., 2021) always precedes pregnancy. In the context of implantation, it is important to emphasise that menstruation is a piecemeal process that involves simultaneous tissue shedding and rapid re-epithelisation of denuded areas, a process that likely involves mesenchymal-epithelial transition (MET) (Garry et al., 2009; Garry et al., 2010). By contrast, endometrial glands are clonal (Moore et al., 2020), and regenerate from basal glands that extend horizontally along the junctional zone (Yamaguchi et al., 2022). Hence, the provenance of the luminal epithelium (the site of initial embryo–maternal contact) differs from glandular epithelium. Further, single-cell transcriptomics have demonstrated that cells expressing both epithelial and mesenchymal marker genes are present in mid-secretory phase endometrium, suggesting that repair of the luminal epithelium upon embryo implantation also involves MET (Lucas et al., 2020).

#### Proliferation phase

**Fig. 4.**
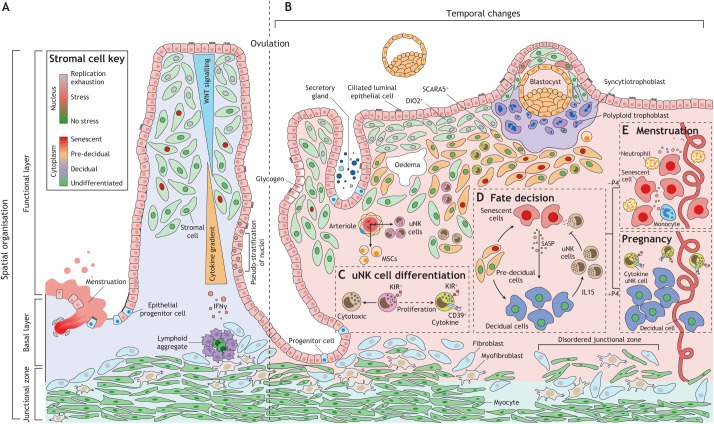
**Cellular dynamics in the endometrium before and during embryo implantation.** (A) Endometrial regeneration leads to cell specification and tissue patterning. While oestradiol drives rapid endometrial growth prior to ovulation, its actions are modified by morphogen and cytokine gradients emanating from the luminal epithelium and lymphoid aggregates in the basal layer, respectively. Consequently, proliferative activity is positional, resulting in the emergence of exhausted, stressed and non-stressed subpopulations as indicated by the colour of nuclei. Morphogen gradients and cell–cell signalling also lead to specification of epithelial cells, exemplified by the emergence of ciliated cells. Rapid growth accounts for the pseudostratified appearance of glands. (B) Following the post-ovulatory rise in progesterone, the endometrium undergoes sequential morphological changes, starting with the appearance of subnuclear ‘vacuoles’ in glandular epithelium in the early secretory phase. The mid-secretory window of implantation coincides with a decidual reaction, characterised by apocrine glandular secretion, stromal oedema and accumulation of uterine natural killer (uNK) cells and bone marrow-derived mesenchymal stem cells (MSCs). Subluminal DIO2^+^ stromal cells are progesterone resistant whereas underlying progesterone responsive (SCARA5^+^) stromal cells decidualise (pre-decidual cells). (C) Differentiation of uNK cells into functionally distinct subpopulations. For detailed explanation, see text. KIR, killer cell immunoglobulin-like receptors. (D) Pre-decidual cells emerge as decidual cells upon closure of the implantation window, although some cells damaged by replication stress give rise to decidual-like senescent cells producing a complex inflammatory secretome (senescence associated secretory phenotype, SASP). The SASP induces secondary senescence in decidual cells, a process countered by decidual cells through activation of uNK cells that target and eliminate stressed/senescent cells. (E) In the late-secretory phase, falling progesterone (−P4) levels lead to expansion of decidual-like senescent cells, influx of neutrophils and monocytes, and menstruation, whereas sustained progesterone signalling (+P4) upon implantation transforms the endometrium into the decidua of pregnancy.

Following menstruation, rising ovarian oestradiol levels lead to rapid growth of the endometrium, which on average quadruples in thickness and volume prior to ovulation (Figs 3B, 4A) (Raine-Fenning et al., 2004a). The rate of endometrial growth over these 10 days is arguably unparalleled in any other tissue. Proliferation of endometrial cells involves activation of multiple growth factor signalling pathways (Fang et al., 2022), accelerates with increasing distance from the basal layer, and peaks around cycle day 10 in the upper third of the endometrium (Ferenczy et al., 1979), coinciding with maximal endometrial vascular perfusion and transient tissue oedema (Raine-Fenning et al., 2004b; Noyes et al., 2019). Depending on the position within the tissue, endometrial cells are subjected to different levels of replication stress (Fig. 4A). In the upper superficial layer, some stromal and epithelial cells express p16^INK4a^ (CDKN2A) and p21^CIP1^ (CDKN1A) (Lv et al., 2022), cyclin-dependent kinase inhibitors commonly used to identify senescent cells damaged by replication exhaustion (Munoz-Espin and Serrano, 2014). Notably, expansion of p16^INK4a^- and p21^CIP1^-positive cells in proliferative phase stroma is associated with oestrogen resistance, lack of endometrial growth and a pathologically thin endometrium (Lv et al., 2022).

Lymphoid aggregates that reside in the basal endometrial layer are believed to regulate the spatial responsiveness of endometrium to ovarian hormones (Christian et al., 2001; Tabibzadeh, 1991; Tabibzadeh et al., 1993). These aggregates comprise several hundred cells and consist of a core of B cells surrounded by T cells and macrophages (Fig. 4A) (Yeaman et al., 1997). Because of their relatively inaccessible location, lymphoid aggregates are poorly characterised, although there is evidence that they are established in each cycle from trafficked circulating immune cells (Yeaman et al., 2001). Further, IFNγ, secreted by activated T cells in the aggregates, is a potent inhibitor of cell proliferation and oestrogen and progesterone signalling (Christian et al., 2001; Tabibzadeh, 1991; Tabibzadeh et al., 1993). As cells progressively escape this inhibitory cytokine gradient, proliferation accelerates and hormone responsiveness increases, thereby restricting cyclical tissue remodelling to the superficial layer (Fig. 4A).

Alongside positional proliferation, morphogen gradients and membrane-bound cell–cell signalling govern cell fate determinations during the proliferative phase (Garcia-Alonso et al., 2021). For example, secreted WNT7A is essential for normal Mullerian tract development (Parr and McMahon, 1998; Roly et al., 2018). In cycling endometrium, its expression is confined to the newly formed luminal epithelium and upper glands during the post-menstrual repair phase (Fan et al., 2012). As the proliferative phase unfolds, expression becomes restricted to the luminal epithelium, thereby creating a WNT gradient (Fig. 4A) (Garcia-Alonso et al., 2021). Activation of the canonical WNT/β-catenin pathway in response to oestradiol promotes ciliogenesis of epithelial cells when cell–cell-dependent NOTCH signalling is low (Haider et al., 2019). However, beyond the WNT microenvironment, high NOTCH activity commits cells to a secretory epithelial phenotype following ovulation (Garcia-Alonso et al., 2021). In development, WNT7A maintains expression of HOXA10 and HOXA11, homeobox transcription factors that provide cells with specific positional identities on the anterior-posterior axis (Roly et al., 2018). Both transcription factors are abundantly expressed in proliferative phase stromal cells (Talbi et al., 2006), indicating that cell fate specification is not confined to the epithelial compartment.

These observations challenge the view that the proliferative phase serves merely to thicken the endometrium in preparation for implantation. Instead, oestradiol activates multiple mechanisms, including intense positional proliferation and morphogen gradients, involved in cell specification and tissue patterning to establish a spatial template on which progesterone will act during the luteal phase (Fig. 4B). Rapid growth of the arteries and angiogenesis, leading to the formation of a subluminal capillary network (Rogers and Gargett, 1998), adds to the complexity of endometrial microenvironments that are established during the proliferative phase. As placentation is deeper in humans than in other primates (Carter et al., 2015), aberrant spatial organisation of the endometrium during the proliferative phase could arguably have disproportional impacts on embryo implantation and pregnancy outcome.

### Endometrial differentiation

#### Early secretory phase

Ovulation leads to a rapid drop in ovarian oestradiol production and rising progesterone levels, which peak 7-8 days later (Johnson et al., 2015). Proliferative activity in the superficial endometrium decreases sharply (Ferenczy et al., 1979), alongside declining WNT7A levels (Fan et al., 2012). Luminal epithelium and abutting glands upregulate prostaglandin-endoperoxide synthase 1 and 2 (PTGS1 and PTGS2), rate-limiting enzymes in the biosynthesis of prostaglandins from arachidonic acid. Whereas PTGS1 is confined to the epithelium, PTGS2 is also upregulated in perivascular stromal cells (Marions and Danielsson, 1999; Garcia-Alonso et al., 2021). In parallel, endometrial prostaglandin concentrations increase following ovulation, characterised first by a steep rise in prostaglandin F2 alpha (PGF2α) levels and then a more modest increase in PGE2 concentrations (Downie et al., 1974). PGF2α is a potent vasoconstrictor and may account for the sudden reduction in endometrial vascular perfusion following ovulation (Raine-Fenning et al., 2004b). Transition to the secretory (luteal) phase also coincides with upregulation of genes encoding metabolic enzymes, transporter proteins and ion channels (Talbi et al., 2006). In luminal epithelium, dynamic changes in ion channel activities lead to progressive absorption of fluid from the uterine cavity, a process that facilitates embryo–endometrial interactions (Ruan et al., 2014; Salker et al., 2011). Cytoplasmic accumulation of glycogen accounts for the appearance of prominent subnuclear ‘vacuoles’ in glandular epithelial cells (Fig. 4B), whereas mitochondrial biogenesis gives rise to ultrastructural ‘giant’ mitochondria (Cornillie et al., 1985). There is also evidence of a metabolic switch in stromal cells, exemplified by rapid progesterone-dependent downregulation of iodothyronine deiodinase 2 (DIO2), which catalyses the conversion of prohormone thyroxine (3,5,3′,5'-tetraiodothyronine, T4) to bioactive thyroid hormone (3,5,3'-triiodothyronine, T3), a master regulator of cellular energy expenditure and basal metabolism (Kim, 2008). However, DIO2 persists in subluminal stromal cells following ovulation (Fig. 4B), marking metabolically active cells that are progesterone resistant and produce an abundance of extracellular matrix (ECM) components (Lucas et al., 2020).

#### Mid-secretory phase/the implantation window

Endometrial gene expression changes abruptly during the mid-secretory (midluteal) phase, reflecting an acute stress response that heralds the start of the implantation window (Wang et al., 2020). This endometrial stress response recapitulates all aspects of a decidual reaction induced by implanting embryos in other mammals, including onset of apocrine glandular secretions, prominent stromal oedema, influx of extra-uterine cells and decidual reprogramming of stromal fibroblasts (Fig. 4B). The emergence of phenotypic decidual cells coincides with the closure of the implantation window and transition to the late luteal phase of the cycle (Noyes et al., 2019; Gellersen and Brosens, 2014). Canonical decidual cells with the ultrastructural appearance of those in pregnancy are most prominent around spiral arterioles (Lawn et al., 1971). These stromal cells have the biophysical properties of pericytes, are often clonogenic, and express selectively vascular adhesion protein 1 (also known as amine oxidase copper containing 3, *AOC3*), suggesting they serve as gatekeepers of infiltrating extra-uterine cells (Gharanei et al., 2021; Lucas et al., 2016; Murakami et al., 2014). Perivascular decidual cells have also been implicated in maintaining tissue haemostasis during the menstrual cycle and upon spiral artery remodelling in pregnancy (Lockwood et al., 2007).

The trigger for the mid-secretory stress response in human endometrium is unclear. In hormonally primed rats and mice, decidualisation can be elicited by non-specific stressors, including intra-uterine oil injection, trauma (scratching or crushing) or electrical stimulation of uterine horns, but only in the presence of a luminal epithelium before and after the insult (Lejeune et al., 1981). In mice, the ability of the luminal epithelium to transduce diverse stress-related inputs into a deciduogenic signal is linked to the presence of protease-activated receptors, including the epithelial sodium receptor (ENaC; SCNN1) (Ruan et al., 2012). Although these evolutionarily conserved receptors are also activated by human blastocyst-derived serine proteases and elicit Ca^2+^-dependent gene expression (Shmygol and Brosens, 2021; Brosens et al., 2014), they cannot account for spontaneous decidualisation in non-conception cycles. A likely explanation for the endogenous deciduogenic signal in human endometrium is the cumulative impact of stress signals associated with rapid tissue growth and vascular changes. Endometrial perfusion increases markedly during the midluteal phase (Raine-Fenning et al., 2004b), thus imposing modest oxidative stress on a small number of cells already damaged by replication stress in the preceding proliferative phase. This may be sufficient to trigger the release of inflammatory mediators and alarmins, including IL33, IL1A and high-mobility group box 1 protein (HMGB1), which in turn propagate the pro-inflammatory response across the tissue (Salker et al., 2012; von Zglinicki et al., 2000). Notably, menstruating species in the primate lineage all lost the ability to synthesise endogenous ascorbic acid (vitamin C), a major antioxidant and important for collagen synthesis and tensile strength (Drouin et al., 2011; Hiller et al., 2012). Hence, it is plausible that relaxation of the ECM combined with reduced cellular antioxidant defences created a permissive environment for the emergence of an endogenous deciduogenic signal by simultaneously enhancing stromal proliferation (and replication stress) and cellular susceptibility to oxidative stress.

Prominent mid-secretory stromal oedema (Fig. 4B) results from transluminal fluid absorption, increased vascular permeability, the near absence of lymphatic vessels in superficial human endometrium (Cornillie et al., 1985; Rogers et al., 2009), and rapid deposition of hyaluronan (Salamonsen et al., 2001). Hyaluronan, a major ECM component, is intimately involved in rapid tissue remodelling and repair. As a high molecular weight polymer, it binds water very efficiently, thereby regulating the viscoelasticity and stiffness of tissues (Amorim et al., 2021). However, breakdown by hyaluronidases generates low molecular weight hyaluronan polymers, which regulate proliferation, migration and activity of immune and other cells upon binding of various receptors, including CD44. Endometrial oedema typically separates subluminal DIO2-positive stromal cells from underlying decidualising stromal cells (pre-decidual cells) that express progesterone-dependent marker genes, such as *SCARA5* (Fig. 4B) (Lucas et al., 2020). As mentioned, a hallmark of decidualisation is accumulation of uNK cells (Catalini and Fedder, 2020), which rapidly outnumber other endometrial immune cells, including T cells, B cells, macrophages and dendritic cells. A recent study involving uterine transplant recipients demonstrated that uNK cells are replenished from the circulation (Strunz et al., 2021). They represent the only proliferating immune cell population in mid-secretory endometrium and differentiate in response to local cues into functionally distinct subsets (Fig. 4C). How uNK cells diversify into subsets in the endometrium remains contentious. One study concluded that uNK cells acquire sequentially killer cell immunoglobulin-like receptors (KIRs) and the ectonucleotidase CD39 (ENTPD1) as the cycle progresses, marking loss of proliferative capacity and a switch from a pro-inflammatory to a cytokine-producing and angiogenic phenotype (Strunz et al., 2021). However, this linear differentiation trajectory of uNK subsets has been challenged by single-cell transcriptomic data (Guo et al., 2021). Further, spatial mapping in early gestation demonstrated that cytokine-producing KIR^+^CD39^+^ uNK cells, which highly express P16^INK4a^ and resemble senescent immune cells (Rajagopalan and Long, 2012), reside in the upper decidua where they engage placental cells through binding of KIRs to a limited repertoire of HLA class I molecules expressed by extravillous trophoblast. By contrast, cytotoxic KIR^−^CD39^−^ uNK cells are found deeper in the tissue (Wang et al., 2021).

### Late secretory phase: menstruation versus pregnancy

The emergence of decidual cells upon closure of the implantation window represents an inflection point in the menstrual cycle after which the endometrium either breaks down or is transformed into the decidua of pregnancy (Gellersen and Brosens, 2014). Insights into the mechanisms that control fate decisions in late secretory phase endometrium has emerged from primary 2D and 3D cultures, which revealed that decidualising endometrial stromal cells not only give rise to anti-inflammatory decidual cells but also decidual-like senescent cells that are targeted and eliminated by activated cytotoxic uNK cells in a progesterone-dependent manner (Fig. 4D) (Brighton et al., 2017; Lucas et al., 2020; Rawlings et al., 2021).

#### Decidual cells

*In vivo*, adenylyl cyclase activity rises in secretory endometrium in parallel with increasing tissue concentrations of the second messenger cyclic adenosine monophosphate (cAMP) (Tanaka et al., 1993; Pansini et al., 1984). In culture, cAMP-dependent protein kinase A (PKA) activation triggers an acute stress response in endometrial stromal cells, characterised by a burst of reactive oxygen species (ROS) production and release of IL1A, HMGB1 and IL33 and other inflammatory mediators (Al-Sabbagh et al., 2011; Brighton et al., 2017; Salker et al., 2012). PKA signalling also activates evolutionarily conserved decidual transcription factors, including CEBPB, FOXO1, GATA2 and HAND2, which in turn leads to sequential regulation of distinct transcriptional programmes (Erkenbrack et al., 2018; Gellersen and Brosens, 2014). Several decidual transcription factors physically engage the liganded progesterone receptor and bind as multimeric complexes to primate-specific TEs and other *cis*-regulatory DNA elements in promoter and enhancer regions of target genes (Christian et al., 2002; Vrljicak et al., 2018; Mika and Lynch, 2022). Hence, progesterone is essential for maintaining decidual gene expression, although insufficient for initiating this differentiation process (Brosens et al., 1999). As is the case *in vivo*, the temporal changes in gene expression in decidualising primary cultures cease after approximately 4 days and anti-inflammatory decidual cells emerge (Lucas et al., 2020). Compared with their progenitors, decidual cells are remarkably resistant to oxidative and metabolic stressors, reflecting progesterone-dependent silencing of multiple stress-activated signalling pathways and increased free radical scavenging capacity (Leitao et al., 2010; Kajihara et al., 2006; Muter et al., 2018). Further, decidual cells become extensively connected by gap junctions, which aids the formation of a decidual bed in pregnancy (Winterhager and Kidder, 2015).

#### Decidual-like senescence cells

As is the case in the endometrium, primary cultures harbour stromal cells damaged by replication stress during proliferative expansion (Brighton et al., 2017). Although only a discrete population, pre-senescent cells are largely responsible for the acute release of inflammatory mediators at the start of the decidual pathway, but subsequently fail to differentiate and emerge after 4 days as decidual-like senescent cells (Lucas et al., 2020; Rawlings et al., 2021). Decidual and canonical senescent cells share multiple characteristics, including cell cycle arrest, expression of survival genes, heightened autonomy from environmental cues, and rounded appearance with abundant cytoplasm and enlarged nuclei (Brighton et al., 2017). However, decidual-like senescent cells are progesterone insensitive and produce a complex secretome, termed senescence-associated secretory phenotype (SASP), rich in ECM proteins and proteinases, growth factors, chemokines, angiogenic factors and inflammatory mediators (Fig. 4D) (Rawlings et al., 2021). Notably, whereas the acute inflammatory response produced by pre-senescent cells at the start of the decidual pathway promotes de-differentiation and clonogenicity of stromal cells (Brighton et al., 2017), decidual-like senescent cells induce bystander (paracrine) senescence in neighbouring decidual cells, resulting in rapid propagation of sterile inflammation, even under conditions of continuous progesterone signalling (Brighton et al., 2017; Lucas et al., 2020; Rawlings et al., 2021). Thus, the default outcome of spontaneous decidualisation is endometrial inflammation and ECM breakdown. Further, senescent cells recruit neutrophils, which in turn reinforces paracrine senescence through ROS production (Lagnado et al., 2021). In human endometrium, macrophages and neutrophil granulocytes accumulate immediately prior to menstruation in a stroma abounding with decidual-like senescent cells (Fig. 4E, top) (Evans and Salamonsen, 2012; Noyes et al., 2019).

Notably, cell state divergence is also apparent in secretory glands, which harbour P16^INK4a^-positive epithelial cells during the implantation window. Although accounting for only 2-3% of cells (Brighton et al., 2017), P16^INK4a^-positive epithelial cells secrete key implantation factors that are also canonical SASP components, including dipeptidyl peptidase 4 (DPP4), growth differentiation factor 15 (GDF15) and insulin-like growth factor binding protein 3 (IGFBP3) (Rawlings et al., 2021).

#### Decidual–uNK cell cooperation

To avoid menstrual tissue breakdown, decidual cells must engage cytotoxic uNK cells. Even before they emerge as fully differentiated decidual cells, progesterone-dependent pre-decidual cells selectively secrete factors involved in uNK cell recruitment, activation and recognition of stressed/senescent cells, including CXCL14, IL15 and TIMP metallopeptidase inhibitor 3 (TIMP3), respectively (Lucas et al., 2020). In primary co-cultures, uNK cells isolated from mid-secretory endometrial biopsies employ granule exocytosis to kill emerging decidual-like senescent cells with total precision and without a need for exogenous priming (Brighton et al., 2017; Kong et al., 2021; Lucas et al., 2020). Similarly, prolonged decidualisation leads to disintegration of endometrial assembloids, 3D cultures that consist of gland-like organoids and primary stromal cells (Rawlings et al., 2021). However, pre-treatment of assembloids with dasatinib, a broad-spectrum tyrosine kinase inhibitor that eliminates pre-senescent/stressed stromal cells, generates exceptionally sturdy decidualised assembloids (Rawlings et al., 2021).

By co-opting uNK cells, pre-decidual cells rejuvenate the endometrium during the implantation window. Further, decidualisation in both humans and mice leads to recruitment of BM-MSCs, which compensate for cell attrition, bestow plasticity on the tissue, and give rise to a distinct subset of prolactin-producing decidual cells in pregnancy (Diniz-da-Costa et al., 2021; Tal et al., 2019). The dependency of decidual–uNK cell cooperation on continuous progesterone signalling imposes a bivalent state on the midluteal endometrium (Fig. 4D), which, upon successful implantation and corpus luteum rescue, result in the formation of the decidua of pregnancy (Fig. 4E, bottom). In non-conception cycles, however, declining progesterone levels in the late secretory phase first gradually reverse expression of progesterone-induced and -repressed genes and and then disable decidual–uNK cell cooperation, which is followed by the sudden demise of uNK cells (Moffett and Shreeve, 2022), a steep rise in P16^INK4^-positive stromal and epithelial cells (Brighton et al., 2017), and influx of senescence-associated neutrophils and macrophages (Evans and Salamonsen, 2012). Once initiated, these processes render menstrual breakdown inevitable. Hence, the window of opportunity for implanting human embryos to rescue ovarian progesterone production and reverse the default trajectory of the decidualising endometrium towards tissue destruction is very narrow. In an experimental primate model, menstruation becomes unavoidable if progesterone is withdrawn for 36 h or longer (Slayden and Brenner, 2006). In humans, the incidence of early pregnancy loss increases exponentially with each day that implantation is delayed beyond the midluteal implantation window (Wilcox et al., 1999).

### Interstitial embryo implantation

Mammalian implantation involves a concatenation of discrete steps, starting with closure of the uterine cavity, hatching of the embryo through the surrounding zona pellucida, orientation and attachment of the conceptus to the apical surface of the luminal epithelium, and invasion of the underlying endometrial stroma (Wang and Dey, 2006). It should be noted that the inferred mechanism of embryo implantation in humans differs substantially from other mammals, such as mice (Box 3). Unfortunately, *in situ* observations of human embryo implantation are confined to historical samples in which the blastocyst is already embedded in the stroma with the overlying luminal epithelium almost repaired (Hertig et al., 1956). Hence, our understanding of the epithelial phase of the implantation process is based on extrapolations from animal studies, or derived from simple *in vitro* models, such as co-culturing surplus IVF embryos donated for research on top of a monolayer of Ishikawa cells, an endometrial adenocarcinoma cell line (Aplin and Ruane, 2017; Aberkane et al., 2018; Ruane et al., 2022). The development of complex 3D endometrial culture systems, such as assembloids, is a promising approach to the study of human embryo implantation (Rawlings et al., 2021), although it remains challenging to simulate the spatial organisation of human endometrium. Another exciting development is the ability to generate human blastoids from naïve pluripotent stem cells (Terhune et al., 2022; Kagawa et al., 2022). Blastoids not only recapitulate embryonic cell fate specifications, but this technology overcomes the lack of scalability and reproducibility inherent to the use of chromosomally diverse human embryos (Yu et al., 2022 preprint).

**Box 3. Implantation in mice**

The inferred mechanism of embryo implantation in humans differs substantially from other mammals, such as mice. Mice are litter-bearing mammals and a robust barrier function of the luminal epithelium is important to ensure synchronised implantation of multiple blastocysts. Murine blastocysts in the uterine cavity can arrest temporarily in development, known as diapause (Box 4), while awaiting a maternal nidation signal. This signal, a finely calibrated rise in maternal oestrogen levels, renders the murine endometrium receptive, activates dormant embryos, and enables adherence of trophectoderm away from the inner cell mass (mural trophectoderm) to the apical surface of the luminal epithelium (Fig. 2B). Embryo attachment then triggers local mucosal inflammation, which in turn initiates the decidual stromal reaction and is followed by shallow mural trophectoderm invasion (Ma et al., 2003). Although oestradiol also rises transiently during the midluteal phase of the menstrual cycle (Fig. 3A), there is no evidence that it serves as a nidation signal (Ghosh et al., 1994; Smitz et al., 1993). Further, comparative single-cell transcriptomic analysis demonstrated that the implantation poles of human and mouse blastocysts (polar and mural trophectoderm, respectively) share only a limited number of enriched genes (Liu et al., 2022).

**Box 4. Embryo–endometrial communication: diapause and biosensing**

Implantation depends on bi-directional communication between the blastocyst and endometrium. Encoded in this communication is information on maternal and embryonic fitness, which in turn can lead to activation of species-specific reproductive suppression mechanisms. For example, blastocysts in over 130 mammals, although not humans, respond to the presence or absence of specific endocrine or endometrial cues by entering or exiting diapause, a state of suspended animation characterised by complete or near-complete cessation of cell division (Renfree and Fenelon, 2017). Depending on the species, implantation can be postponed for days or months and serves to either maximise the number of offspring in each season or synchronise parturition with environmental conditions favourable to offspring survival. By contrast, embryo biosensing depends on the endometrium receiving and decoding fitness information from the conceptus. Genome-wide expression profiling demonstrated that, depending on their fitness, bovine embryos elicit distinct endometrial transcriptomic responses predictive of subsequent pregnancy outcome (Bauersachs et al., 2009; Mansouri-Attia et al., 2009). The prominence of a given mechanism of reproductive suppression in a species does not necessarily imply loss or redundancy of other mechanisms. For example, upon transfer into mice uteri, sheep blastocysts, which do not exhibit diapause, become growth arrested and quiescent under induced diapause conditions, resuming development when subsequently placed into sheep uteri (Ptak et al., 2012). Similarly, flushing the mouse uterus with spent culture medium from low-fitness human IVF embryos triggers an endometrial stress response, whereas the medium of successfully implanted human embryos upregulates endometrial metabolic and implantation genes. Remarkably, cues from competent human embryos strongly induce the expression of murine-specific, implantation-specific genes (*Prss28* and *Prss29*), which are lost in humans and other primates (Brosens et al., 2014). These observations suggest that evolutionarily conserved, rather than acquired, mechanisms underpin the exchange of fitness information between individual embryos and the endometrium.

Despite the limitations of current *in vitro* systems, important insights into epithelial phase of the implantation process have emerged. The pre-implantation human blastocyst consists of an outer trophectoderm layer that surrounds the inner cell mass and a fluid-filled cavity, the blastocoel. Trophectoderm cells are the precursors of placental lineages, whereas inner cell mass cells give rise to the pluripotent epiblast and primitive endoderm, precursors of the embryo proper and yolk sac, respectively (Meistermann et al., 2021). Around the time of hatching, epiblast signals promote differentiation of the adjacent trophectoderm, termed polar trophectoderm, leading to loss of cell cycle genes and induction of primitive syncytiotrophoblast markers, including human endogenous retrovirus (HERV) genes that are essential for trophoblast fusion and placental development (Liu et al., 2022; Ruane et al., 2022; Lv et al., 2019). Consequently, human embryos orient such that the polar trophectoderm attaches to the endometrial epithelium. In co-culture studies, attachment upregulates adhesion-related genes in trophectoderm cells, accelerates their differentiation into invasive multinuclear primitive syncytiotrophoblast, enhances hCG secretion and kickstarts progesterone production (Liu et al., 2022; Ruane et al., 2022). Although multiple candidate adhesion molecules and receptor–ligand pairs have been implicated in embryo–epithelium interactions, a precise understanding of the molecular events in the epithelial phase of the implantation process is still lacking.

A notable observation in co-culture experiments is that human blastocysts often penetrate epithelial monolayers seemingly without much effort. Decidualising stromal cells in 2D and 3D cultures also migrate rapidly to co-cultured human blastocysts with individual cells appearing to compete for attachment to the polar trophectoderm before pulling the conceptus into the endometrial matrix (Berkhout et al., 2018; Weimar et al., 2012; Rawlings et al., 2021). Pharmacological depletion of pre-senescent/stressed cells in endometrial assembloids massively accelerates the emergence of anti-inflammatory decidual cells, which in turn entrap co-cultured human embryos into a robust but static environment devoid of blastocyst–stromal cell interactions (Rawlings et al., 2021). Together, these observations cast doubt on the assumption that breaching of the luminal epithelium by the conceptus is the rate-limiting step in the implantation process and, by extension, the point of implantation failure. Instead, interstitial embryo implantation seems to rely on close cooperation between epithelial cells and underlying DIO2-positive stromal cells, resulting in a transient epithelial attachment phase, which is followed by rapid encapsulation of the entire conceptus by decidualising stromal cells. Human embryos also secrete hyaluronidase 2 (HYAL2) (Kong et al., 2021), which plausibly promotes interstitial implantation by destabilising oedema associated with high molecular weight hyaluronan.

### The implantation checkpoint: embryo biosensing and selection

**Fig. 5.**
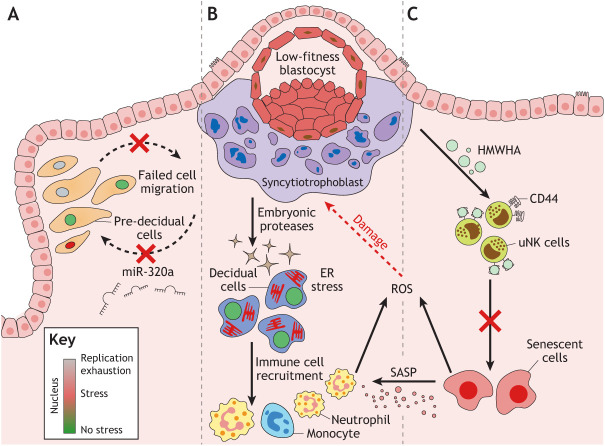
**Natural selection of human embryos at implantation.** Multiple mechanisms in decidualising endometrium ensure rapid elimination of low-fitness embryos. (A) Pre-decidual cells migrate and actively encapsulate embryos that breached the luminal epithelium. This migratory response is disrupted upon implantation of a poor-quality embryo, which has been linked to the lack of embryonic secretion of hsa-miR-320a. (B) Proteases secreted by low-fitness embryos induce prolonged and disordered Ca^2+^ signalling in pre-decidual cells, causing endoplasmic reticulum (ER) stress, attenuated secretion of implantation factors and induction of chemokines involved in recruitment of neutrophils and monocytes. (C) Low-fitness embryos secrete high molecular weight hyaluronic acid (HMWHA), which, upon binding to CD44 on uNK cells, blocks targeting and elimination of stressed/senescent cells, thereby causing sterile tissue inflammation through secondary senescence and menstruation-like breakdown of the endometrium, irrespective of circulating progesterone levels. Further, reactive oxygen species (ROS) produced by decidual-like senescent cells and immune cells may damage the conceptus and impair or preclude further development.

A pivotal aspect of the implantation process in all mammals is exchange of fitness information between the mother and conceptus (Box 4). Compelling evidence of this phenomenon in humans emerged from *in vitro* migration assays that modelled interstitial implantation (Macklon and Brosens, 2014). These studies revealed that undifferentiated endometrial stromal cells migrate to low-quality human blastocysts, as assessed by morphological criteria, but not high-quality embryos. Decidualisation enhances the migratory capacity of stromal cells. However, in contrast to their undifferentiated counterparts, decidualising cells migrate selectively to high- but not low-quality embryos (Berkhout et al., 2018; Weimar et al., 2012). How decidualisation switches the behaviour of stromal cells to embryonic fitness signals is unclear, although selective migration to high-quality blastocysts has been linked to embryonic secretion of hsa-miR-320a (MIR320A), an evolutionarily conserved microRNA essential for pre-implantation embryo development (Fig. 5A) (Berkhout et al., 2020; Feng et al., 2015). Further, endometrial epithelial cells and decidualising stromal cells are exquisitely responsive to serine proteases produced by low-quality embryos and mount an endoplasmic stress response that silences secretion of pivotal implantation factors, including HB-EGF (HBEGF) and LIF (Fig. 5B) (Teklenburg et al., 2010; Brosens et al., 2014; Shmygol and Brosens, 2021). More recently, spent medium of good-quality IVF blastocysts that either implanted successfully or not was used to explore how implantation could trigger a menstruation-like reaction, even in patients receiving progesterone therapy (Kong et al., 2021). In co-culture experiments, medium conditioned by unsuccessful embryos completely abrogated uNK cell-mediated killing of senescent cells, whereas spent medium from successful embryos had no impact. Loss of immune surveillance of decidual-like senescent cells was attributed to lack of HYAL2 activity in unsuccessful embryos and binding of high molecular weight hyaluronan to the ECM receptor CD44 on uNK cells (Fig. 5C) (Kong et al., 2021). There is also evidence that exposure of decidualising cells to conditioned medium from low-quality IVF blastocysts increases IL8 (CXCL8) secretion and promotes recruitment and activation of neutrophils (Fernández et al., 2022). Conversely, ROS secreted by decidual-like senescent cells may damage trophoblast and limit invasion (Fig. 5C) (Deryabin et al., 2022).

Thus, multiple strands of evidence suggest that low-fitness embryos upend the poised endometrial state during the implantation window by activating mechanisms that will lead to tissue destruction. By contrast, soluble signals produced by high-quality embryos likely impact on the decidual transformation of the endometrium (Box 4). In this context, several studies focussed on hCG signalling in decidualising cells, but the findings are inconsistent (Mann et al., 2022). However, hCG does induce uNK cell proliferation (Kane et al., 2009), which promotes the emergence of cytokine producing KIR^+^CD39^+^ uNK cells involved in vascular remodelling (Wang et al., 2021). Clinically, embryo biosensing followed by physiological elimination of low-fitness embryos can account for the high incidence of occult pregnancy losses in young healthy women, the age-dependent decline in fertility rates, and the limited implantation rates in IVF (Brosens et al., 2022; Pirtea et al., 2021). However, fitness impacts of genetic errors in human embryos are origin dependent and chromosome specific, meaning that blastocysts that harbour certain aneuploidies, such as meiotic trisomies of smaller chromosomes, could plausibly escape the implantation checkpoint, thus explaining the age-dependent increase in clinical aneuploid pregnancy losses (Box 2) (Brosens et al., 2022).

## Concluding remarks

Once considered a black box, our understanding of the mechanisms that control human embryo implantation is accelerating rapidly, aided by powerful new technologies including single-cell ‘omics’, spatial transcriptomics, blastoids, organoids and assembloids. Although much is yet to be discovered, the notion that human reproduction is inefficient is pertinently wrong; in view of the challenges posed by chromosomal instability in embryos, human reproduction should be considered strategic, malleable and ruthlessly selective. Although the basic principles of pregnancy in humans are shared with all mammals, the combination of interstitial implantation, deep haemochorial placentation and dependence on robust embryo selection in great apes are underpinned by novel reproductive traits, including intense spatial and temporal remodelling of the endometrium and junctional zone myometrium in response to oestradiol and progesterone signalling, respectively. Perhaps the most surprising aspect of these human-specific reproductive traits is that intrinsic chromosome instability in embryos is countered by cellular processes in the endometrium with strong pathological connotations, including replication stress, DNA damage, cellular senescence, inflammation and tissue destruction. However, as outlined in this Review, these processes in cycling endometrium also imbue the implantation process with exceptional plasticity, exemplified clinically by high cumulative live birth rates.

## References

[DEV201507C1] Abbas, Y., Carnicer-Lombarte, A., Gardner, L., Thomas, J., Brosens, J. J., Moffett, A., Sharkey, A. M., Franze, K., Burton, G. J. and Oyen, M. L. (2019). Tissue stiffness at the human maternal-fetal interface. *Hum. Reprod.* 34, 1999-2008. 10.1093/humrep/dez139 31579915 PMC6809602

[DEV201507C2] Aberkane, A., Essahib, W., Spits, C., De Paepe, C., Sermon, K., Adriaenssens, T., Mackens, S., Tournaye, H., Brosens, J. J. and Van De Velde, H. (2018). Expression of adhesion and extracellular matrix genes in human blastocysts upon attachment in a 2D co-culture system. *Mol. Hum. Reprod.* 24, 375-387. 10.1093/molehr/gay024 29846687

[DEV201507C3] Al-Sabbagh, M., Fusi, L., Higham, J., Lee, Y., Lei, K., Hanyaloglu, A. C., Lam, E. W., Christian, M. and Brosens, J. J. (2011). NADPH oxidase-derived reactive oxygen species mediate decidualization of human endometrial stromal cells in response to cyclic AMP signaling. *Endocrinology* 152, 730-740. 10.1210/en.2010-0899 21159852 PMC3037160

[DEV201507C4] Amorim, S., Reis, C. A., Reis, R. L. and Pires, R. A. (2021). Extracellular matrix mimics using hyaluronan-based biomaterials. *Trends Biotechnol.* 39, 90-104. 10.1016/j.tibtech.2020.06.003 32654775

[DEV201507C5] Aplin, J. D. and Ruane, P. T. (2017). Embryo-epithelium interactions during implantation at a glance. *J. Cell Sci.* 130, 15-22. 10.1242/jcs.175943 28043966

[DEV201507C6] Bauersachs, S., Ulbrich, S. E., Zakhartchenko, V., Minten, M., Reichenbach, M., Reichenbach, H. D., Blum, H., Spencer, T. E. and Wolf, E. (2009). The endometrium responds differently to cloned versus fertilized embryos. *Proc. Natl. Acad. Sci. USA* 106, 5681-5686. 10.1073/pnas.0811841106 19307558 PMC2666995

[DEV201507C7] Beehner, J. C. and Lu, A. (2013). Reproductive suppression in female primates: a review. *Evol. Anthropol.* 22, 226-238. 10.1002/evan.21369 24166923

[DEV201507C8] Bellofiore, N., Cousins, F., Temple-Smith, P., Dickinson, H. and Evans, J. (2018). A missing piece: the spiny mouse and the puzzle of menstruating species. *J. Mol. Endocrinol.* 61, R25-R41. 10.1530/JME-17-0278 29789322

[DEV201507C9] Berkhout, R. P., Lambalk, C. B., Huirne, J., Mijatovic, V., Repping, S., Hamer, G. and Mastenbroek, S. (2018). High-quality human preimplantation embryos actively influence endometrial stromal cell migration. *J. Assist. Reprod. Genet.* 35, 659-667. 10.1007/s10815-017-1107-z 29282583 PMC5949101

[DEV201507C10] Berkhout, R. P., Keijser, R., Repping, S., Lambalk, C. B., Afink, G. B., Mastenbroek, S. and Hamer, G. (2020). High-quality human preimplantation embryos stimulate endometrial stromal cell migration via secretion of microRNA hsa-miR-320a. *Hum. Reprod.* 35, 1797-1807. 10.1093/humrep/deaa149 32644109 PMC7398623

[DEV201507C11] Bolton, H., Graham, S. J. L., Van Der Aa, N., Kumar, P., Theunis, K., Fernandez Gallardo, E., Voet, T. and Zernicka-Goetz, M. (2016). Mouse model of chromosome mosaicism reveals lineage-specific depletion of aneuploid cells and normal developmental potential. *Nat. Commun.* 7, 11165. 10.1038/ncomms11165 27021558 PMC4820631

[DEV201507C12] Bortoletto, P., Lucas, E. S., Melo, P., Gallos, I. D., Devall, A. J., Bourne, T., Quenby, S., Bennett, P. R., Coomarasamy, A. and Brosens, J. J. (2022). Miscarriage syndrome: linking early pregnancy loss to obstetric and age-related disorders. *EBioMedicine* 81, 104134. 10.1016/j.ebiom.2022.104134 35779492 PMC9244729

[DEV201507C13] Brighton, P. J., Maruyama, Y., Fishwick, K., Vrljicak, P., Tewary, S., Fujihara, R., Muter, J., Lucas, E. S., Yamada, T., Woods, L. et al. (2017). Clearance of senescent decidual cells by uterine natural killer cells in cycling human endometrium. *eLife* 6, e31274. 10.7554/eLife.31274 29227245 PMC5724991

[DEV201507C14] Brosens, J. J., De Souza, N. M. and Barker, F. G. (1995). Uterine junctional zone: function and disease. *Lancet* 346, 558-560. 10.1016/S0140-6736(95)91387-4 7658784

[DEV201507C15] Brosens, J. J., Barker, F. G. and De Souza, N. M. (1998). Myometrial zonal differentiation and uterine junctional zone hyperplasia in the non-pregnant uterus. *Hum. Reprod. Update* 4, 496-502. 10.1093/humupd/4.5.496 10027601

[DEV201507C16] Brosens, J. J., Hayashi, N. and White, J. O. (1999). Progesterone receptor regulates decidual prolactin expression in differentiating human endometrial stromal cells. *Endocrinology* 140, 4809-4820. 10.1210/endo.140.10.7070 10499541

[DEV201507C17] Brosens, J. J., Pijnenborg, R. and Brosens, I. A. (2002). The myometrial junctional zone spiral arteries in normal and abnormal pregnancies: a review of the literature. *Am. J. Obstet. Gynecol.* 187, 1416-1423. 10.1067/mob.2002.127305 12439541

[DEV201507C18] Brosens, J. J., Tullet, J., Varshochi, R. and Lam, E. W. (2004). Steroid receptor action. *Best Pract. Res. Clin. Obstet. Gynaecol.* 18, 265-283. 10.1016/j.bpobgyn.2004.01.006 15157642

[DEV201507C19] Brosens, J. J., Parker, M. G., Mcindoe, A., Pijnenborg, R. and Brosens, I. A. (2009). A role for menstruation in preconditioning the uterus for successful pregnancy. *Am. J. Obstet. Gynecol.* 200, 615.e1-6. 10.1016/j.ajog.2008.11.037 19136085

[DEV201507C20] Brosens, J. J., Salker, M. S., Teklenburg, G., Nautiyal, J., Salter, S., Lucas, E. S., Steel, J. H., Christian, M., Chan, Y. W., Boomsma, C. M. et al. (2014). Uterine selection of human embryos at implantation. *Sci. Rep.* 4, 3894. 10.1038/srep03894 24503642 PMC3915549

[DEV201507C21] Brosens, I., Muter, J., Gargett, C. E., Puttemans, P., Benagiano, G. and Brosens, J. J. (2017). The impact of uterine immaturity on obstetrical syndromes during adolescence. *Am. J. Obstet. Gynecol.* 217, 546-555. 10.1016/j.ajog.2017.05.059 28578177

[DEV201507C22] Brosens, J. J., Bennett, P. R., Abrahams, V. M., Ramhorst, R., Coomarasamy, A., Quenby, S., Lucas, E. S. and Mccoy, R. C. (2022). Maternal selection of human embryos in early gestation: insights from recurrent miscarriage. *Semin. Cell Dev. Biol.* 131, 14-24. 10.1016/j.semcdb.2022.01.007 35094946 PMC9325922

[DEV201507C23] Bull, J. R., Rowland, S. P., Scherwitzl, E. B., Scherwitzl, R., Danielsson, K. G. and Harper, J. (2019). Real-world menstrual cycle characteristics of more than 600,000 menstrual cycles. *NPJ Digit. Med.* 2, 83. 10.1038/s41746-019-0152-7 31482137 PMC6710244

[DEV201507C24] Carter, A. M. and Enders, A. C. (2004). Comparative aspects of trophoblast development and placentation. *Reprod. Biol. Endocrinol.* 2, 46. 10.1186/1477-7827-2-46 15236656 PMC455692

[DEV201507C25] Carter, A. M., Enders, A. C. and Pijnenborg, R. (2015). The role of invasive trophoblast in implantation and placentation of primates. *Philos. Trans. R. Soc. Lond. B Biol. Sci.* 370, 20140070. 10.1098/rstb.2014.0070 25602074 PMC4305171

[DEV201507C26] Casarini, L., Santi, D., Brigante, G. and Simoni, M. (2018). Two hormones for one receptor: evolution, biochemistry, actions, and pathophysiology of LH and hCG. *Endocr. Rev.* 39, 549-592. 10.1210/er.2018-00065 29905829

[DEV201507C27] Catalini, L. and Fedder, J. (2020). Characteristics of the endometrium in menstruating species: lessons learned from the animal kingdomdagger. *Biol. Reprod.* 102, 1160-1169. 10.1093/biolre/ioaa029 32129461 PMC7253787

[DEV201507C28] Chavan, A. R., Griffith, O. W., Stadtmauer, D. J., Maziarz, J., Pavlicev, M., Fishman, R., Koren, L., Romero, R. and Wagner, G. P. (2021). Evolution of embryo implantation was enabled by the origin of decidual stromal cells in eutherian mammals. *Mol. Biol. Evol.* 38, 1060-1074. 10.1093/molbev/msaa274 33185661 PMC7947829

[DEV201507C29] Christian, M., Marangos, P., Mak, I., Mcvey, J., Barker, F., White, J. and Brosens, J. J. (2001). Interferon-gamma modulates prolactin and tissue factor expression in differentiating human endometrial stromal cells. *Endocrinology* 142, 3142-3151. 10.1210/endo.142.7.8231 11416037

[DEV201507C30] Christian, M., Zhang, X., Schneider-Merck, T., Unterman, T. G., Gellersen, B., White, J. O. and Brosens, J. J. (2002). Cyclic AMP-induced forkhead transcription factor, FKHR, cooperates with CCAAT/enhancer-binding protein beta in differentiating human endometrial stromal cells. *J. Biol. Chem.* 277, 20825-20832. 10.1074/jbc.M201018200 11893744

[DEV201507C31] Cornillie, F. J., Lauweryns, J. M. and Brosens, I. A. (1985). Normal human endometrium. An ultrastructural survey. *Gynecol. Obstet. Invest.* 20, 113-129. 10.1159/000298983 4085915

[DEV201507C32] Coughlan, C., Ledger, W., Wang, Q., Liu, F., Demirol, A., Gurgan, T., Cutting, R., Ong, K., Sallam, H. and Li, T. C. (2014). Recurrent implantation failure: definition and management. *Reprod. Biomed. Online* 28, 14-38. 10.1016/j.rbmo.2013.08.011 24269084

[DEV201507C33] Cousins, F. L., Filby, C. E. and Gargett, C. E. (2022). Endometrial stem/progenitor cells-their role in endometrial repair and regeneration. *Front. Reprod. Health* 3, 811537. 10.3389/frph.2021.811537 36304009 PMC9580754

[DEV201507C34] Craciunas, L., Gallos, I., Chu, J., Bourne, T., Quenby, S., Brosens, J. J. and Coomarasamy, A. (2019). Conventional and modern markers of endometrial receptivity: a systematic review and meta-analysis. *Hum. Reprod. Update* 25, 202-223. 10.1093/humupd/dmy044 30624659

[DEV201507C35] Crespo, Â. C., Mulik, S., Dotiwala, F., Ansara, J. A., Sen Santara, S., Ingersoll, K., Ovies, C., Junqueira, C., Tilburgs, T., Strominger, J. L. et al. (2020). Decidual NK cells transfer granulysin to selectively kill bacteria in trophoblasts. *Cell* 182, 1125-1139.e18. 10.1016/j.cell.2020.07.019 32822574 PMC7484179

[DEV201507C36] Critchley, H. O. D., Babayev, E., Bulun, S. E., Clark, S., Garcia-Grau, I., Gregersen, P. K., Kilcoyne, A., Kim, J. J., Lavender, M., Marsh, E. E. et al. (2020). Menstruation: science and society. *Am. J. Obstet. Gynecol.* 223, 624-664. 10.1016/j.ajog.2020.06.004 32707266 PMC7661839

[DEV201507C37] Csapo, A. I., Pulkkinen, M. O. and Wiest, W. G. (1973). Effects of luteectomy and progesterone replacement therapy in early pregnant patients. *Am. J. Obstet. Gynecol.* 115, 759-765. 10.1016/0002-9378(73)90517-6 4688578

[DEV201507C38] Deryabin, P. I., Ivanova, J. S. and Borodkina, A. V. (2022). Senescent endometrial stromal cells transmit reactive oxygen species to the trophoblast-like cells and impair spreading of blastocyst-like spheroids. *Mol. Hum. Reprod.* 28, gaac039. 10.1093/molehr/gaac039 36370081

[DEV201507C39] Diniz-Da-Costa, M., Kong, C. S., Fishwick, K. J., Rawlings, T., Brighton, P. J., Hawkes, A., Odendaal, J., Quenby, S., Ott, S., Lucas, E. S. et al. (2021). Characterization of highly proliferative decidual precursor cells during the window of implantation in human endometrium. *Stem Cells* 39, 1067-1080. 10.1002/stem.3367 33764639

[DEV201507C40] Downie, J., Poyser, N. L. and Wunderlich, M. (1974). Levels of prostaglandins in human endometrium during the normal menstrual cycle. *J. Physiol.* 236, 465-472. 10.1113/jphysiol.1974.sp010446 16992446 PMC1350813

[DEV201507C41] Drouin, G., Godin, J. R. and Pagé, B. (2011). The genetics of vitamin C loss in vertebrates. *Curr. Genomics* 12, 371-378. 10.2174/138920211796429736 22294879 PMC3145266

[DEV201507C42] Dunson, D. B., Colombo, B. and Baird, D. D. (2002). Changes with age in the level and duration of fertility in the menstrual cycle. *Hum. Reprod.* 17, 1399-1403. 10.1093/humrep/17.5.1399 11980771

[DEV201507C43] Elliot, M. G. and Crespi, B. J. (2006). Placental invasiveness mediates the evolution of hybrid inviability in mammals. *Am. Nat.* 168, 114-120. 10.1086/505162 16874618

[DEV201507C44] Emera, D., Romero, R. and Wagner, G. (2012). The evolution of menstruation: a new model for genetic assimilation: explaining molecular origins of maternal responses to fetal invasiveness. *BioEssays* 34, 26-35. 10.1002/bies.201100099 22057551 PMC3528014

[DEV201507C45] Erkenbrack, E. M., Maziarz, J. D., Griffith, O. W., Liang, C., Chavan, A. R., Nnamani, M. C. and Wagner, G. P. (2018). The mammalian decidual cell evolved from a cellular stress response. *PLoS Biol.* 16, e2005594. 10.1371/journal.pbio.2005594 30142145 PMC6108454

[DEV201507C46] Evans, J. and Salamonsen, L. A. (2012). Inflammation, leukocytes and menstruation. *Rev. Endocr. Metab. Disord.* 13, 277-288. 10.1007/s11154-012-9223-7 22865231

[DEV201507C47] Evers, J. L. (2002). Female subfertility. *Lancet* 360, 151-159. 10.1016/S0140-6736(02)09417-5 12126838

[DEV201507C48] Fan, X., Krieg, S., Hwang, J. Y., Dhal, S., Kuo, C. J., Lasley, B. L., Brenner, R. M. and Nayak, N. R. (2012). Dynamic regulation of Wnt7a expression in the primate endometrium: implications for postmenstrual regeneration and secretory transformation. *Endocrinology* 153, 1063-1069. 10.1210/en.2011-1826 22294752 PMC3281546

[DEV201507C49] Fang, Z., Tian, Y., Sui, C., Guo, Y., Hu, X., Lai, Y., Liao, Z., Li, J., Feng, G., Jin, L. et al. (2022). Single-cell transcriptomics of proliferative phase endometrium: systems analysis of cell-cell communication network using CellChat. *Front. Cell Dev. Biol.* 10, 919731. 10.3389/fcell.2022.919731 35938159 PMC9352955

[DEV201507C50] Feng, R., Sang, Q., Zhu, Y., Fu, W., Liu, M., Xu, Y., Shi, H., Xu, Y., Qu, R., Chai, R. et al. (2015). MiRNA-320 in the human follicular fluid is associated with embryo quality in vivo and affects mouse embryonic development in vitro. *Sci. Rep.* 5, 8689. 10.1038/srep08689 25732513 PMC4346788

[DEV201507C51] Ferenczy, A., Bertrand, G. and Gelfand, M. M. (1979). Proliferation kinetics of human endometrium during the normal menstrual cycle. *Am. J. Obstet. Gynecol.* 133, 859-867. 10.1016/0002-9378(79)90302-8 434029

[DEV201507C52] Fernández, L., Grasso, E., Soczewski, E., Gori, S., Calo, G., Hauk, V., Sabbione, F., Gallino, L., Martínez, G., Irigoyen, M. et al. (2022). Understanding the natural selection of human embryos: blastocyst quality modulates the inflammatory response during the peri-implantation period. *Am. J. Reprod. Immunol.* 87, e13423. 10.1111/aji.13423 33764560

[DEV201507C53] Finn, C. A. (1998). Menstruation: a nonadaptive consequence of uterine evolution. *Q. Rev. Biol.* 73, 163-173. 10.1086/420183 9618925

[DEV201507C54] Foo, L., Johnson, S., Marriott, L., Bourne, T., Bennett, P. and Lees, C. (2020). Peri-implantation urinary hormone monitoring distinguishes between types of first-trimester spontaneous pregnancy loss. *Paediatr. Perinat. Epidemiol.* 34, 495-503. 10.1111/ppe.12613 32056241 PMC7496486

[DEV201507C55] Fujii, S., Konishi, I. and Mori, T. (1989). Smooth muscle differentiation at endometrio-myometrial junction. An ultrastructural study. *Virchows Arch. A Pathol. Anat. Histopathol.* 414, 105-112. 10.1007/BF00718589 2492689

[DEV201507C56] Garcia-Alonso, L., Handfield, L. F., Roberts, K., Nikolakopoulou, K., Fernando, R. C., Gardner, L., Woodhams, B., Arutyunyan, A., Polanski, K., Hoo, R. et al. (2021). Mapping the temporal and spatial dynamics of the human endometrium in vivo and in vitro. *Nat. Genet.* 53, 1698-1711. 10.1038/s41588-021-00972-2 34857954 PMC8648563

[DEV201507C57] Garry, R., Hart, R., Karthigasu, K. A. and Burke, C. (2009). A re-appraisal of the morphological changes within the endometrium during menstruation: a hysteroscopic, histological and scanning electron microscopic study. *Hum. Reprod.* 24, 1393-1401. 10.1093/humrep/dep036 19252193

[DEV201507C58] Garry, R., Hart, R., Karthigasu, K. A. and Burke, C. (2010). Structural changes in endometrial basal glands during menstruation. *BJOG* 117, 1175-1185. 10.1111/j.1471-0528.2010.02630.x 20560946

[DEV201507C59] Gellersen, B. and Brosens, J. J. (2014). Cyclic decidualization of the human endometrium in reproductive health and failure. *Endocr. Rev.* 35, 851-905. 10.1210/er.2014-1045 25141152

[DEV201507C60] Gharanei, S., Fishwick, K., Peter Durairaj, R., Jin, T., Siamantouras, E., Liu, K. K., Straube, A., Lucas, E. S., Weston, C. J., Rantakari, P. et al. (2021). Vascular adhesion protein-1 determines the cellular properties of endometrial pericytes. *Front. Cell Dev. Biol.* 8, 621016. 10.3389/fcell.2020.621016 33537312 PMC7848099

[DEV201507C61] Ghosh, D., De, P. and Sengupta, J. (1994). Luteal phase ovarian oestrogen is not essential for implantation and maintenance of pregnancy from surrogate embryo transfer in the rhesus monkey. *Hum. Reprod.* 9, 629-637. 10.1093/oxfordjournals.humrep.a138561 8046014

[DEV201507C62] Gobert, M. and Lafaille, J. J. (2012). Maternal-fetal immune tolerance, block by block. *Cell* 150, 7-9. 10.1016/j.cell.2012.06.020 22770210 PMC4061910

[DEV201507C63] Greco, E., Minasi, M. G. and Fiorentino, F. (2015). Healthy babies after intrauterine transfer of mosaic aneuploid blastocysts. *N. Engl. J. Med.* 373, 2089-2090. 10.1056/NEJMc1500421 26581010

[DEV201507C64] Griffith, O. W., Chavan, A. R., Protopapas, S., Maziarz, J., Romero, R. and Wagner, G. P. (2017). Embryo implantation evolved from an ancestral inflammatory attachment reaction. *Proc. Natl. Acad. Sci. USA* 114, E6566-E6575. 10.1073/pnas.1701129114 28747528 PMC5559003

[DEV201507C65] Gruhn, J. R., Zielinska, A. P., Shukla, V., Blanshard, R., Capalbo, A., Cimadomo, D., Nikiforov, D., Chan, A. C.-H., Newnham, L. J. and Vogel, I. et al. (2019). Chromosome errors in human eggs shape natural fertility over reproductive life span. *Science* 365, 1466-1469. 10.1126/science.aav7321 31604276 PMC7212007

[DEV201507C66] Guo, C., Cai, P., Jin, L., Sha, Q., Yu, Q., Zhang, W., Jiang, C., Liu, Q., Zong, D., Li, K. et al. (2021). Single-cell profiling of the human decidual immune microenvironment in patients with recurrent pregnancy loss. *Cell Discov.* 7, 1. 10.1038/s41421-020-00236-z 33390590 PMC7779601

[DEV201507C67] Haider, S., Gamperl, M., Burkard, T. R., Kunihs, V., Kaindl, U., Junttila, S., Fiala, C., Schmidt, K., Mendjan, S., Knöfler, M. et al. (2019). Estrogen signaling drives ciliogenesis in human endometrial organoids. *Endocrinology* 160, 2282-2297. 10.1210/en.2019-00314 31290979

[DEV201507C68] Haig, D. (1993). Genetic conflicts in human pregnancy. *Q. Rev. Biol.* 68, 495-532. 10.1086/418300 8115596

[DEV201507C69] Haig, D. (2019). Cooperation and conflict in human pregnancy. *Curr. Biol.* 29, R455-R458. 10.1016/j.cub.2019.04.040 31163157

[DEV201507C70] Hansen, V. L., Schilkey, F. D. and Miller, R. D. (2016). Transcriptomic changes associated with pregnancy in a marsupial, the gray short-tailed opossum Monodelphis domestica. *PLoS One* 11, e0161608. 10.1371/journal.pone.0161608 27598793 PMC5012577

[DEV201507C71] Hardy, K., Hardy, P. J., Jacobs, P. A., Lewallen, K. and Hassold, T. J. (2016). Temporal changes in chromosome abnormalities in human spontaneous abortions: results of 40 years of analysis. *Am. J. Med. Genet. A* 170, 2671-2680. 10.1002/ajmg.a.37795 27287007

[DEV201507C72] Hertig, A. and Bernischke, K. (1967). *Comparative Aspects of Reproductive Failure*. Editado por Bermischke K. Editorial Springer-Verlag New York.

[DEV201507C73] Hertig, A. T., Rock, J. and Adams, E. C. (1956). A description of 34 human ova within the first 17 days of development. *Am. J. Anat.* 98, 435-493. 10.1002/aja.1000980306 13362122

[DEV201507C74] Hiller, M., Schaar, B. T., Indjeian, V. B., Kingsley, D. M., Hagey, L. R. and Bejerano, G. (2012). A ‘forward genomics’ approach links genotype to phenotype using independent phenotypic losses among related species. *Cell Rep.* 2, 817-823. 10.1016/j.celrep.2012.08.032 23022484 PMC3572205

[DEV201507C75] Huhn, O., Ivarsson, M. A., Gardner, L., Hollinshead, M., Stinchcombe, J. C., Chen, P., Shreeve, N., Chazara, O., Farrell, L. E., Theorell, J. et al. (2020). Distinctive phenotypes and functions of innate lymphoid cells in human decidua during early pregnancy. *Nat. Commun.* 11, 381. 10.1038/s41467-019-14123-z 31959757 PMC6971012

[DEV201507C76] Jarvis, G. E. (2016). Early embryo mortality in natural human reproduction: what the data say. *F1000Res* 5, 2765. 10.12688/f1000research.8937.1 28580126 PMC5443340

[DEV201507C77] Johnson, S., Weddell, S., Godbert, S., Freundl, G., Roos, J. and Gnoth, C. (2015). Development of the first urinary reproductive hormone ranges referenced to independently determined ovulation day. *Clin. Chem. Lab. Med.* 53, 1099-1108. 10.1515/cclm-2014-1087 25720077

[DEV201507C78] Kagawa, H., Javali, A., Khoei, H. H., Sommer, T. M., Sestini, G., Novatchkova, M., Scholte Op Reimer, Y., Castel, G., Bruneau, A., Maenhoudt, N., et al. (2022). Human blastoids model blastocyst development and implantation. *Nature* 601, 600-605. 10.1038/s41586-021-04267-8 34856602 PMC8791832

[DEV201507C79] Kahn, D. A. and Baltimore, D. (2010). Pregnancy induces a fetal antigen-specific maternal T regulatory cell response that contributes to tolerance. *Proc. Natl. Acad. Sci. USA* 107, 9299-9304. 10.1073/pnas.1003909107 20439708 PMC2889122

[DEV201507C80] Kajihara, T., Jones, M., Fusi, L., Takano, M., Feroze-Zaidi, F., Pirianov, G., Mehmet, H., Ishihara, O., Higham, J. M., Lam, E. W. et al. (2006). Differential expression of FOXO1 and FOXO3a confers resistance to oxidative cell death upon endometrial decidualization. *Mol. Endocrinol.* 20, 2444-2455. 10.1210/me.2006-0118 16709600

[DEV201507C81] Kajihara, T., Tanaka, K., Oguro, T., Tochigi, H., Prechapanich, J., Uchino, S., Itakura, A., Sućurović, S., Murakami, K., Brosens, J. J. et al. (2014). Androgens modulate the morphological characteristics of human endometrial stromal cells decidualized in vitro. *Reprod. Sci.* 21, 372-380. 10.1177/1933719113497280 23885104

[DEV201507C82] Kane, N., Kelly, R., Saunders, P. T. and Critchley, H. O. (2009). Proliferation of uterine natural killer cells is induced by human chorionic gonadotropin and mediated via the mannose receptor. *Endocrinology* 150, 2882-2888. 10.1210/en.2008-1309 19196802 PMC2709965

[DEV201507C83] Kim, B. (2008). Thyroid hormone as a determinant of energy expenditure and the basal metabolic rate. *Thyroid* 18, 141-144. 10.1089/thy.2007.0266 18279014

[DEV201507C84] Kolte, A. M., Westergaard, D., Lidegaard, O., Brunak, S. and Nielsen, H. S. (2021). Chance of live birth: a nationwide, registry-based cohort study. *Hum. Reprod.* 36, 1065-1073. 10.1093/humrep/deaa326 33394013

[DEV201507C85] Kong, C. S., Ordonez, A. A., Turner, S., Tremaine, T., Muter, J., Lucas, E. S., Salisbury, E., Vassena, R., Tiscornia, G., Fouladi-Nashta, A. A. et al. (2021). Embryo biosensing by uterine natural killer cells determines endometrial fate decisions at implantation. *FASEB J.* 35, e21336. 10.1096/fj.202002217R 33749894 PMC8251835

[DEV201507C86] Konishi, I., Fujii, S., Okamura, H. and Mori, T. (1984). Development of smooth muscle in the human fetal uterus: an ultrastructural study. *J. Anat.* 139, 239-252. 6490516 PMC1164372

[DEV201507C87] Kshitiz, Afzal, J., Maziarz, J. D., Hamidzadeh, A., Liang, C., Erkenbrack, E. M., Kim, H. N., Haeger, J. D., Pfarrer, C., Hoang, T. et al. (2019). Evolution of placental invasion and cancer metastasis are causally linked. *Nat. Ecol. Evol.* 3, 1743-1753. 10.1038/s41559-019-1046-4 31768023 PMC7340496

[DEV201507C88] Kunz, G., Beil, D., Deininger, H., Wildt, L. and Leyendecker, G. (1996). The dynamics of rapid sperm transport through the female genital tract: evidence from vaginal sonography of uterine peristalsis and hysterosalpingoscintigraphy. *Hum. Reprod.* 11, 627-632. 10.1093/HUMREP/11.3.627 8671281

[DEV201507C89] Kunz, G., Noe, M., Herbertz, M. and Leyendecker, G. (1998). Uterine peristalsis during the follicular phase of the menstrual cycle: effects of oestrogen, antioestrogen and oxytocin. *Hum. Reprod. Update* 4, 647-654. 10.1093/humupd/4.5.647 10027618

[DEV201507C90] Lagnado, A., Leslie, J., Ruchaud-Sparagano, M. H., Victorelli, S., Hirsova, P., Ogrodnik, M., Collins, A. L., Vizioli, M. G., Habiballa, L., Saretzki, G. et al. (2021). Neutrophils induce paracrine telomere dysfunction and senescence in ROS-dependent manner. *EMBO J.* 40, e106048. 10.15252/embj.2020106048 33764576 PMC8090854

[DEV201507C91] Lawn, A. M., Wilson, E. W. and Finn, C. A. (1971). The ultrastructure of human decidual and predecidual cells. *J. Reprod. Fertil.* 26, 85-90. 10.1530/jrf.0.0260085 5091297

[DEV201507C92] Leitao, B., Jones, M. C., Fusi, L., Higham, J., Lee, Y., Takano, M., Goto, T., Christian, M., Lam, E. W. and Brosens, J. J. (2010). Silencing of the JNK pathway maintains progesterone receptor activity in decidualizing human endometrial stromal cells exposed to oxidative stress signals. *FASEB J.* 24, 1541-1551. 10.1096/fj.09-149153 20026682 PMC2857868

[DEV201507C93] Lejeune, B., Van Hoeck, J. and Leroy, F. (1981). Transmitter role of the luminal uterine epithelium in the induction of decidualization in rats. *J. Reprod. Fertil.* 61, 235-240. 10.1530/jrf.0.0610235 7452622

[DEV201507C94] Lesny, P., Killick, S. R., Tetlow, R. L., Manton, D. J., Robinson, J. and Maguiness, S. D. (1999). Ultrasound evaluation of the uterine zonal anatomy during *in-vitro* fertilization and embryo transfer. *Hum. Reprod.* 14, 1593-1598. 10.1093/humrep/14.6.1593 10357982

[DEV201507C95] Li, R. and Zhu, J. (2022). Effects of aneuploidy on cell behaviour and function. *Nat. Rev. Mol. Cell Biol.* 23, 250-265. 10.1038/s41580-021-00436-9 34987171

[DEV201507C96] Liu, D., Chen, Y., Ren, Y., Yuan, P., Wang, N., Liu, Q., Yang, C., Yan, Z., Yang, M., Wang, J. et al. (2022). Primary specification of blastocyst trophectoderm by scRNA-seq: new insights into embryo implantation. *Sci. Adv.* 8, eabj3725. 10.1126/sciadv.abj3725 35947672 PMC9365277

[DEV201507C97] Lockwood, C. J., Krikun, G., Rahman, M., Caze, R., Buchwalder, L. and Schatz, F. (2007). The role of decidualization in regulating endometrial hemostasis during the menstrual cycle, gestation, and in pathological states. *Semin. Thromb. Hemost.* 33, 111-117. 10.1055/s-2006-958469 17253197

[DEV201507C98] Lucas, E. S., Dyer, N. P., Murakami, K., Lee, Y. H., Chan, Y. W., Grimaldi, G., Muter, J., Brighton, P. J., Moore, J. D., Patel, G. et al. (2016). Loss of endometrial plasticity in recurrent pregnancy loss. *Stem Cells* 34, 346-356. 10.1002/stem.2222 26418742

[DEV201507C99] Lucas, E. S., Vrljicak, P., Muter, J., Diniz-Da-Costa, M. M., Brighton, P. J., Kong, C. S., Lipecki, J., Fishwick, K. J., Odendaal, J., Ewington, L. J. et al. (2020). Recurrent pregnancy loss is associated with a pro-senescent decidual response during the peri-implantation window. *Commun. Biol.* 3, 37. 10.1038/s42003-020-0763-1 31965050 PMC6972755

[DEV201507C100] Lv, B., An, Q., Zeng, Q., Zhang, X., Lu, P., Wang, Y., Zhu, X., Ji, Y., Fan, G. and Xue, Z. (2019). Single-cell RNA sequencing reveals regulatory mechanism for trophoblast cell-fate divergence in human peri-implantation conceptuses. *PLoS Biol.* 17, e3000187. 10.1371/journal.pbio.3000187 31596842 PMC6802852

[DEV201507C101] Lv, H., Zhao, G., Jiang, P., Wang, H., Wang, Z., Yao, S., Zhou, Z., Wang, L., Liu, D., Deng, W. et al. (2022). Deciphering the endometrial niche of human thin endometrium at single-cell resolution. *Proc. Natl. Acad. Sci. USA* 119, e2115912119. 10.1073/pnas.2115912119 35169075 PMC8872762

[DEV201507C102] Lynch, V. J., Leclerc, R. D., May, G. and Wagner, G. P. (2011). Transposon-mediated rewiring of gene regulatory networks contributed to the evolution of pregnancy in mammals. *Nat. Genet.* 43, 1154-1159. 10.1038/ng.917 21946353

[DEV201507C103] Lynch, V. J., Nnamani, M. C., Kapusta, A., Brayer, K., Plaza, S. L., Mazur, E. C., Emera, D., Sheikh, S. Z., Grutzner, F., Bauersachs, S. et al. (2015). Ancient transposable elements transformed the uterine regulatory landscape and transcriptome during the evolution of mammalian pregnancy. *Cell Rep.* 10, 551-561. 10.1016/j.celrep.2014.12.052 25640180 PMC4447085

[DEV201507C104] Ma, W. G., Song, H., Das, S. K., Paria, B. C. and Dey, S. K. (2003). Estrogen is a critical determinant that specifies the duration of the window of uterine receptivity for implantation. *Proc. Natl. Acad. Sci. USA* 100, 2963-2968. 10.1073/pnas.0530162100 12601161 PMC151449

[DEV201507C105] Macklon, N. S. and Brosens, J. J. (2014). The human endometrium as a sensor of embryo quality. *Biol. Reprod.* 91, 98. 10.1095/biolreprod.114.122846 25187529

[DEV201507C106] Macklon, N. S., Geraedts, J. P. and Fauser, B. C. (2002). Conception to ongoing pregnancy: the ‘black box’ of early pregnancy loss. *Hum. Reprod. Update* 8, 333-343. 10.1093/humupd/8.4.333 12206468

[DEV201507C107] Magnus, M. C., Wilcox, A. J., Morken, N. H., Weinberg, C. R. and Haberg, S. E. (2019). Role of maternal age and pregnancy history in risk of miscarriage: prospective register based study. *BMJ* 364, l869. 10.1136/bmj.l869 30894356 PMC6425455

[DEV201507C108] Male, V. (2021). Medawar and the immunological paradox of pregnancy: in context. *Oxf. Open Immunol.* 2, iqaa006. 10.1093/oxfimm/iqaa006 36845570 PMC9914476

[DEV201507C109] Mann, O. N., Kong, C. S., Lucas, E. S., Brosens, J. J., Hanyaloglu, A. C. and Brighton, P. J. (2022). Expression and function of the luteinizing hormone choriogonadotropin receptor in human endometrial stromal cells. *Sci. Rep.* 12, 8624. 10.1038/s41598-022-12495-9 35597810 PMC9124191

[DEV201507C110] Mansouri-Attia, N., Sandra, O., Aubert, J., Degrelle, S., Everts, R. E., Giraud-Delville, C., Heyman, Y., Galio, L., Hue, I., Yang, X. et al. (2009). Endometrium as an early sensor of in vitro embryo manipulation technologies. *Proc. Natl. Acad. Sci. USA* 106, 5687-5692. 10.1073/pnas.0812722106 19297625 PMC2667091

[DEV201507C111] Marions, L. and Danielsson, K. G. (1999). Expression of cyclo-oxygenase in human endometrium during the implantation period. *Mol. Hum. Reprod.* 5, 961-965. 10.1093/molehr/5.10.961 10508225

[DEV201507C112] McCoy, R. C., Demko, Z. P., Ryan, A., Banjevic, M., Hill, M., Sigurjonsson, S., Rabinowitz, M. and Petrov, D. A. (2015). Evidence of selection against complex mitotic-origin aneuploidy during preimplantation development. *PLoS Genet.* 11, e1005601. 10.1371/journal.pgen.1005601 26491874 PMC4619652

[DEV201507C113] McCoy, R. C., Summers, M. C., McCollin, A., Ottolini, C. S. and Handyside, A. (2022). Meiotic and mitotic aneuploidies drive arrest of in vitro fertilized human preimplantation embryos. *Fertil. Steril*. 118, e76. 10.1016/j.fertnstert.2022.08.233 PMC10544495 37779206

[DEV201507C114] Meistermann, D., Bruneau, A., Loubersac, S., Reignier, A., Firmin, J., François-Campion, V., Kilens, S., Lelièvre, Y., Lammers, J., Feyeux, M. et al. (2021). Integrated pseudotime analysis of human pre-implantation embryo single-cell transcriptomes reveals the dynamics of lineage specification. *Cell Stem Cell* 28, 1625-1640.e6. 10.1016/j.stem.2021.04.027 34004179

[DEV201507C115] Mesiano, S. (2022). Progesterone withdrawal and parturition. *J. Steroid Biochem. Mol. Biol.* 224, 106177. 10.1016/j.jsbmb.2022.106177 36096351

[DEV201507C116] Mika, K. and Lynch, V. J. (2022). Transposable elements continuously remodel the regulatory landscape, transcriptome, and function of decidual stromal cells. *Genome Biol. Evol.* 14, evac164. 10.1093/gbe/evac164 36423206 PMC9732941

[DEV201507C117] Mika, K., Marinic, M., Singh, M., Muter, J., Brosens, J. J. and Lynch, V. J. (2021). Evolutionary transcriptomics implicates new genes and pathways in human pregnancy and adverse pregnancy outcomes. *eLife* 10, e69584. 10.7554/eLife.69584 34623259 PMC8660021

[DEV201507C118] Mika, K., Whittington, C. M., Mcallan, B. M. and Lynch, V. J. (2022). Gene expression phylogenies and ancestral transcriptome reconstruction resolves major transitions in the origins of pregnancy. *eLife* 11, e74297. 10.7554/eLife.74297 35770963 PMC9275820

[DEV201507C119] Moffett, A. and Shreeve, N. (2022). Local immune recognition of trophoblast in early human pregnancy: controversies and questions. *Nat. Rev. Immunol.* 23, 222-235. 10.1038/s41577-022-00777-2 36192648 PMC9527719

[DEV201507C120] Moore, L., Leongamornlert, D., Coorens, T. H. H., Sanders, M. A., Ellis, P., Dentro, S. C., Dawson, K. J., Butler, T., Rahbari, R., Mitchell, T. J. et al. (2020). The mutational landscape of normal human endometrial epithelium. *Nature* 580, 640-646. 10.1038/s41586-020-2214-z 32350471

[DEV201507C121] Munoz-Espin, D. and Serrano, M. (2014). Cellular senescence: from physiology to pathology. *Nat. Rev. Mol. Cell Biol.* 15, 482-496. 10.1038/nrm3823 24954210

[DEV201507C122] Murakami, K., Lee, Y. H., Lucas, E. S., Chan, Y. W., Durairaj, R. P., Takeda, S., Moore, J. D., Tan, B. K., Quenby, S., Chan, J. K. et al. (2014). Decidualization induces a secretome switch in perivascular niche cells of the human endometrium. *Endocrinology* 155, 4542-4553. 10.1210/en.2014-1370 25116707

[DEV201507C123] Muter, J., Alam, M. T., Vrljicak, P., Barros, F. S. V., Ruane, P. T., Ewington, L. J., Aplin, J. D., Westwood, M. and Brosens, J. J. (2018). The glycosyltransferase EOGT regulates adropin expression in decidualizing human endometrium. *Endocrinology* 159, 994-1004. 10.1210/en.2017-03064 29244071

[DEV201507C124] Naik, S. and Fuchs, E. (2022). Inflammatory memory and tissue adaptation in sickness and in health. *Nature* 607, 249-255. 10.1038/s41586-022-04919-3 35831602 PMC9302602

[DEV201507C125] Norwitz, E. R., Schust, D. J. and Fisher, S. J. (2001). Implantation and the survival of early pregnancy. *N. Engl. J. Med.* 345, 1400-1408. 10.1056/NEJMra000763 11794174

[DEV201507C126] Noyes, R. W., Hertig, A. T. and Rock, J. (2019). Reprint of: dating the endometrial biopsy. *Fertil. Steril.* 112, e93-e115. 10.1016/j.fertnstert.2019.08.079 31623748

[DEV201507C127] Pansini, F., Bergamini, C. M., Bettocchi, S., Jr., Malfaccini, M., Santoiemma, M., Scoppetta, V., Bagni, B. and Mollica, G. (1984). Sex steroid hormones influence the cAMP content in human endometrium during the menstrual cycle. *Gynecol. Obstet. Invest.* 18, 174-177. 10.1159/000299076 6096230

[DEV201507C128] Parr, B. A. and Mcmahon, A. P. (1998). Sexually dimorphic development of the mammalian reproductive tract requires Wnt-7a. *Nature* 395, 707-710. 10.1038/27221 9790192

[DEV201507C129] Philips, E. A., Garcia-España, A., Tocheva, A. S., Ahearn, I. M., Adam, K. R., Pan, R., Mor, A. and Kong, X. P. (2020). The structural features that distinguish PD-L2 from PD-L1 emerged in placental mammals. *J. Biol. Chem.* 295, 4372-4380. 10.1074/jbc.AC119.011747 31882544 PMC7135984

[DEV201507C130] Pijnenborg, R., Bland, J. M., Robertson, W. B., Dixon, G. and Brosens, I. (1981). The pattern of interstitial trophoblastic invasion of the myometrium in early human pregnancy. *Placenta* 2, 303-316. 10.1016/S0143-4004(81)80027-6 7301778

[DEV201507C131] Pirtea, P., De Ziegler, D., Tao, X., Sun, L., Zhan, Y., Ayoubi, J. M., Seli, E., Franasiak, J. M. and Scott, R. T. Jr. (2021). Rate of true recurrent implantation failure is low: results of three successive frozen euploid single embryo transfers. *Fertil. Steril.* 115, 45-53. 10.1016/j.fertnstert.2020.07.002 33077239

[DEV201507C132] Polanski, L. T., Baumgarten, M. N., Quenby, S., Brosens, J., Campbell, B. K. and Raine-Fenning, N. J. (2014). What exactly do we mean by ‘recurrent implantation failure’? A systematic review and opinion. *Reprod. Biomed. Online* 28, 409-423. 10.1016/j.rbmo.2013.12.006 24581986

[DEV201507C133] Popovic, M., Dhaenens, L., Taelman, J., Dheedene, A., Bialecka, M., De Sutter, P., Chuva, D. E., Sousa Lopes, S. M., Menten, B. and Heindryckx, B. (2019). Extended in vitro culture of human embryos demonstrates the complex nature of diagnosing chromosomal mosaicism from a single trophectoderm biopsy. *Hum. Reprod.* 34, 758-769. 10.1093/humrep/dez012 30838420

[DEV201507C134] Ptak, G. E., Tacconi, E., Czernik, M., Toschi, P., Modlinski, J. A. and Loi, P. (2012). Embryonic diapause is conserved across mammals. *PLoS One* 7, e33027. 10.1371/journal.pone.0033027 22427933 PMC3299720

[DEV201507C135] Raine-Fenning, N. J., Campbell, B. K., Clewes, J. S., Kendall, N. R. and Johnson, I. R. (2004a). Defining endometrial growth during the menstrual cycle with three-dimensional ultrasound. *BJOG* 111, 944-949. 10.1111/j.1471-0528.2004.00214.x 15327609

[DEV201507C136] Raine-Fenning, N. J., Campbell, B. K., Kendall, N. R., Clewes, J. S. and Johnson, I. R. (2004b). Endometrial and subendometrial perfusion are impaired in women with unexplained subfertility. *Hum. Reprod.* 19, 2605-2614. 10.1093/humrep/deh459 15465835

[DEV201507C137] Rajagopalan, S. and Long, E. O. (2012). Cellular senescence induced by CD158d reprograms natural killer cells to promote vascular remodeling. *Proc. Natl. Acad. Sci. USA* 109, 20596-20601. 10.1073/pnas.1208248109 23184984 PMC3528503

[DEV201507C138] Ramsey, E. M., Houston, M. L. and Harris, J. W. (1976). Interactions of the trophoblast and maternal tissues in three closely related primate species. *Am. J. Obstet. Gynecol.* 124, 647-652. 10.1016/0002-9378(76)90068-5 816200

[DEV201507C139] Rawlings, T. M., Makwana, K., Taylor, D. M., Mole, M. A., Fishwick, K. J., Tryfonos, M., Odendaal, J., Hawkes, A., Zernicka-Goetz, M., Hartshorne, G. M. et al. (2021). Modelling the impact of decidual senescence on embryo implantation in human endometrial assembloids. *eLife* 10, e69603. 10.7554/eLife.69603 34487490 PMC8523170

[DEV201507C140] Renfree, M. B. and Fenelon, J. C. (2017). The enigma of embryonic diapause. *Development* 144, 3199-3210. 10.1242/dev.148213 28928280

[DEV201507C141] Rizzuto, G., Brooks, J. F., Tuomivaara, S. T., Mcintyre, T. I., Ma, S., Rideaux, D., Zikherman, J., Fisher, S. J. and Erlebacher, A. (2022). Establishment of fetomaternal tolerance through glycan-mediated B cell suppression. *Nature* 603, 497-502. 10.1038/s41586-022-04471-0 35236989 PMC9592526

[DEV201507C142] Rogers, P. A. and Gargett, C. E. (1998). Endometrial angiogenesis. *Angiogenesis* 2, 287-294. 10.1023/A:1009222030539 14517449

[DEV201507C143] Rogers, P. A., Donoghue, J. F., Walter, L. M. and Girling, J. E. (2009). Endometrial angiogenesis, vascular maturation, and lymphangiogenesis. *Reprod. Sci.* 16, 147-151. 10.1177/1933719108325509 19001552

[DEV201507C144] Roly, Z. Y., Backhouse, B., Cutting, A., Tan, T. Y., Sinclair, A. H., Ayers, K. L., Major, A. T. and Smith, C. A. (2018). The cell biology and molecular genetics of Müllerian duct development. *Wiley Interdiscip. Rev. Dev. Biol.* 7, e310. 10.1002/wdev.310 29350886

[DEV201507C145] Rothchild, I. (2003). The yolkless egg and the evolution of eutherian viviparity. *Biol. Reprod.* 68, 337-357. 10.1095/biolreprod.102.004531 12533395

[DEV201507C146] Ruan, Y. C., Guo, J. H., Liu, X., Zhang, R., Tsang, L. L., Dong, J. D., Chen, H., Yu, M. K., Jiang, X., Zhang, X. H. et al. (2012). Activation of the epithelial Na+ channel triggers prostaglandin E₂ release and production required for embryo implantation. *Nat. Med.* 18, 1112-1117. 10.1038/nm.2771 22729284

[DEV201507C147] Ruan, Y. C., Chen, H. and Chan, H. C. (2014). Ion channels in the endometrium: regulation of endometrial receptivity and embryo implantation. *Hum. Reprod. Update* 20, 517-529. 10.1093/humupd/dmu006 24591147

[DEV201507C148] Ruane, P. T., Garner, T., Parsons, L., Babbington, P. A., Wangsaputra, I., Kimber, S. J., Stevens, A., Westwood, M., Brison, D. R. and Aplin, J. D. (2022). Trophectoderm differentiation to invasive syncytiotrophoblast is promoted by endometrial epithelial cells during human embryo implantation. *Hum. Reprod.* 37, 777-792. 10.1093/humrep/deac008 35079788 PMC9398450

[DEV201507C149] Salamonsen, L. A., Shuster, S. and Stern, R. (2001). Distribution of hyaluronan in human endometrium across the menstrual cycle. Implications for implantation and menstruation. *Cell Tissue Res.* 306, 335-340. 10.1007/s004410100452 11702245

[DEV201507C202] Salamonsen, L. A., Hutchison, J. C. and Gargett, C. E. (2021). Cyclical endometrial repair and regeneration. *Development* 148, dev199577. 10.1242/dev.199577 34486650

[DEV201507C150] Salker, M. S., Christian, M., Steel, J. H., Nautiyal, J., Lavery, S., Trew, G., Webster, Z., Al-Sabbagh, M., Puchchakayala, G., Foller, M. et al. (2011). Deregulation of the serum- and glucocorticoid-inducible kinase SGK1 in the endometrium causes reproductive failure. *Nat. Med.* 17, 1509-1513. 10.1038/nm.2498 22001908

[DEV201507C151] Salker, M. S., Nautiyal, J., Steel, J. H., Webster, Z., Sucurovic, S., Nicou, M., Singh, Y., Lucas, E. S., Murakami, K., Chan, Y. W. et al. (2012). Disordered IL-33/ST2 activation in decidualizing stromal cells prolongs uterine receptivity in women with recurrent pregnancy loss. *PLoS One* 7, e52252. 10.1371/journal.pone.0052252 23300625 PMC3531406

[DEV201507C152] Samstein, R. M., Josefowicz, S. Z., Arvey, A., Treuting, P. M. and Rudensky, A. Y. (2012). Extrathymic generation of regulatory T cells in placental mammals mitigates maternal-fetal conflict. *Cell* 150, 29-38. 10.1016/j.cell.2012.05.031 22770213 PMC3422629

[DEV201507C153] Senft, A. D. and Macfarlan, T. S. (2021). Transposable elements shape the evolution of mammalian development. *Nat. Rev. Genet.*. 22, 691-711. 10.1038/s41576-021-00385-1 34354263

[DEV201507C154] Shahbazi, M. N., Wang, T., Tao, X., Weatherbee, B. A. T., Sun, L., Zhan, Y., Keller, L., Smith, G. D., Pellicer, A., Scott, R. T. Jr. et al. (2020). Developmental potential of aneuploid human embryos cultured beyond implantation. *Nat. Commun.* 11, 3987. 10.1038/s41467-020-17764-7 32778678 PMC7418029

[DEV201507C155] Shmygol, A. and Brosens, J. J. (2021). Proteinase activated receptors mediate the trypsin-induced Ca^2+^ signaling in human uterine epithelial cells. *Front. Cell Dev. Biol.* 9, 709902. 10.3389/fcell.2021.709902 34434932 PMC8381647

[DEV201507C156] Shoham, Z., Schacter, M., Loumaye, E., Weissman, A., Macnamee, M. and Insler, V. and Insler, V. (1995). The luteinizing hormone surge--the final stage in ovulation induction: modern aspects of ovulation triggering. *Fertil. Steril.* 64, 237-251. 10.1016/S0015-0282(16)57717-6 7615097

[DEV201507C157] Siewiera, J., Mcintyre, T. I., Cautivo, K. M., Mahiddine, K., Rideaux, D., Molofsky, A. B. and Erlebacher, A. (2023). Circumvention of luteolysis reveals parturition pathways in mice dependent upon innate type 2 immunity. *Immunity* 56, 606-619.e7. 10.1016/j.immuni.2023.01.005 36750100 PMC10023352

[DEV201507C158] Singla, S., Iwamoto-Stohl, L. K., Zhu, M. and Zernicka-Goetz, M. (2020). Autophagy-mediated apoptosis eliminates aneuploid cells in a mouse model of chromosome mosaicism. *Nat. Commun.* 11, 2958. 10.1038/s41467-020-16796-3 32528010 PMC7290028

[DEV201507C159] Slayden, O. D. and Brenner, R. M. (2006). A critical period of progesterone withdrawal precedes menstruation in macaques. *Reprod. Biol. Endocrinol.* 4 Suppl 1, S6. 10.1186/1477-7827-4-S1-S6 17118170 PMC1775066

[DEV201507C160] Smith, A., Tilling, K., Nelson, S. M. and Lawlor, D. A. (2015). Live-birth rate associated with repeat in vitro fertilization treatment cycles. *JAMA* 314, 2654-2662. 10.1001/jama.2015.17296 26717030 PMC4934614

[DEV201507C161] Smitz, J., Bourgain, C., Van Waesberghe, L., Camus, M., Devroey, P. and Van Steirteghem, A. C. (1993). A prospective randomized study on oestradiol valerate supplementation in addition to intravaginal micronized progesterone in buserelin and HMG induced superovulation. *Hum. Reprod.* 8, 40-45. 10.1093/oxfordjournals.humrep.a137871 8458924

[DEV201507C162] Soares, M. J., Varberg, K. M. and Iqbal, K. (2018). Hemochorial placentation: development, function, and adaptations. *Biol. Reprod.* 99, 196-211. 10.1093/biolre/ioy049 29481584 PMC6044390

[DEV201507C163] Stadtmauer, D. J. and Wagner, G. P. (2020a). Cooperative inflammation: the recruitment of inflammatory signaling in marsupial and eutherian pregnancy. *J. Reprod. Immunol.* 137, 102626. 10.1016/j.jri.2019.102626 31783286 PMC7028515

[DEV201507C164] Stadtmauer, D. J. and Wagner, G. P. (2020b). The primacy of maternal innovations to the evolution of embryo implantation. *Integr. Comp. Biol.* 60, 742-752. 10.1093/icb/icaa030 32525521 PMC7546962

[DEV201507C165] Starostik, M. R., Sosina, O. A. and McCoy, R. C. (2020). Single-cell analysis of human embryos reveals diverse patterns of aneuploidy and mosaicism. *Genome Res.* 30, 814-825. 10.1101/gr.262774.120 32641298 PMC7370883

[DEV201507C166] Strunz, B., Bister, J., Jonsson, H., Filipovic, I., Crona-Guterstam, Y., Kvedaraite, E., Sleiers, N., Dumitrescu, B., Brannstrom, M., Lentini, A. et al. (2021). Continuous human uterine NK cell differentiation in response to endometrial regeneration and pregnancy. *Sci. Immunol.* 6, eabb7800. 10.1126/sciimmunol.abb7800 33617461

[DEV201507C167] Suhail, Y., Maziarz, J. D., Novin, A., Dighe, A., Afzal, J., Wagner, G. and Kshitiz, (2022). Tracing the cis-regulatory changes underlying the endometrial control of placental invasion. *Proc. Natl. Acad. Sci. USA* 119, e2111256119. 10.1073/pnas.2111256119 35110402 PMC8832988

[DEV201507C168] Tabibzadeh, S. (1991). Induction of HLA-DR expression in endometrial epithelial cells by endometrial T-cells: potential regulatory role of endometrial T-cells in vivo. *J. Clin. Endocrinol. Metab.* 73, 1352-1359. 10.1210/jcem-73-6-1352 1720127

[DEV201507C169] Tabibzadeh, S., Sun, X. Z., Kong, Q. F., Kasnic, G., Miller, J. and Satyaswaroop, P. G. (1993). Induction of a polarized micro-environment by human T cells and interferon-gamma in three-dimensional spheroid cultures of human endometrial epithelial cells. *Hum. Reprod.* 8, 182-192. 10.1093/oxfordjournals.humrep.a138020 8473417

[DEV201507C170] Tal, R., Shaikh, S., Pallavi, P., Tal, A., Lopez-Giraldez, F., Lyu, F., Fang, Y. Y., Chinchanikar, S., Liu, Y., Kliman, H. J. et al. (2019). Adult bone marrow progenitors become decidual cells and contribute to embryo implantation and pregnancy. *PLoS Biol.* 17, e3000421. 10.1371/journal.pbio.3000421 31513564 PMC6742226

[DEV201507C171] Talbi, S., Hamilton, A. E., Vo, K. C., Tulac, S., Overgaard, M. T., Dosiou, C., Le Shay, N., Nezhat, C. N., Kempson, R., Lessey, B. A. et al. (2006). Molecular phenotyping of human endometrium distinguishes menstrual cycle phases and underlying biological processes in normo-ovulatory women. *Endocrinology* 147, 1097-1121. 10.1210/en.2005-1076 16306079

[DEV201507C172] Tanaka, N., Miyazaki, K., Tashiro, H., Mizutani, H. and Okamura, H. (1993). Changes in adenylyl cyclase activity in human endometrium during the menstrual cycle and in human decidua during pregnancy. *J. Reprod. Fertil.* 98, 33-39. 10.1530/jrf.0.0980033 8345477

[DEV201507C173] Teklenburg, G., Salker, M., Molokhia, M., Lavery, S., Trew, G., Aojanepong, T., Mardon, H. J., Lokugamage, A. U., Rai, R., Landles, C. et al. (2010). Natural selection of human embryos: decidualizing endometrial stromal cells serve as sensors of embryo quality upon implantation. *PLoS One* 5, e10258. 10.1371/journal.pone.0010258 20422011 PMC2858159

[DEV201507C174] Terhune, A. H., Bok, J., Sun, S. and Fu, J. (2022). Stem cell-based models of early mammalian development. *Development* 149, dev201015. 10.1242/dev.201015 36255368 PMC10655920

[DEV201507C175] Thurber, C., Dugas, L. R., Ocobock, C., Carlson, B., Speakman, J. R. and Pontzer, H. (2019). Extreme events reveal an alimentary limit on sustained maximal human energy expenditure. *Sci. Adv.* 5, eaaw0341. 10.1126/sciadv.aaw0341 31183404 PMC6551185

[DEV201507C176] Tongsong, T. and Boonyanurak, P. (2004). Placental thickness in the first half of pregnancy. *J. Clin. Ultrasound* 32, 231-234. 10.1002/jcu.20023 15124189

[DEV201507C177] Turnbull, L. W., Manton, D. J., Horsman, A. and Killick, S. R. (1995). Magnetic resonance imaging changes in uterine zonal anatomy during a conception cycle. *Br. J. Obstet. Gynaecol.* 102, 330-331. 10.1111/j.1471-0528.1995.tb09141.x 7612518

[DEV201507C178] Vento-Tormo, R., Efremova, M., Botting, R. A., Turco, M. Y., Vento-Tormo, M., Meyer, K. B., Park, J. E., Stephenson, E., Polanski, K., Goncalves, A. et al. (2018). Single-cell reconstruction of the early maternal-fetal interface in humans. *Nature* 563, 347-353. 10.1038/s41586-018-0698-6 30429548 PMC7612850

[DEV201507C179] Viotti, M., Victor, A. R., Barnes, F. L., Zouves, C. G., Besser, A. G., Grifo, J. A., Cheng, E. H., Lee, M. S., Horcajadas, J. A., Corti, L. et al. (2021). Using outcome data from one thousand mosaic embryo transfers to formulate an embryo ranking system for clinical use. *Fertil. Steril.* 115, 1212-1224. 10.1016/j.fertnstert.2020.11.041 33685629

[DEV201507C180] Vivier, E., Tomasello, E., Baratin, M., Walzer, T. and Ugolini, S. (2008). Functions of natural killer cells. *Nat. Immunol.* 9, 503-510. 10.1038/ni1582 18425107

[DEV201507C181] Von Zglinicki, T., Pilger, R. and Sitte, N. (2000). Accumulation of single-strand breaks is the major cause of telomere shortening in human fibroblasts. *Free Radic. Biol. Med.* 28, 64-74. 10.1016/S0891-5849(99)00207-5 10656292

[DEV201507C182] Vrljicak, P., Lucas, E. S., Lansdowne, L., Lucciola, R., Muter, J., Dyer, N. P., Brosens, J. J. and Ott, S. (2018). Analysis of chromatin accessibility in decidualizing human endometrial stromal cells. *FASEB J.* 32, 2467-2477. 10.1096/fj.201701098R 29259032 PMC6040682

[DEV201507C183] Wang, H. and Dey, S. K. (2006). Roadmap to embryo implantation: clues from mouse models. *Nat. Rev. Genet.* 7, 185-199. 10.1038/nrg1808 16485018

[DEV201507C184] Wang, X., Chen, C., Wang, L., Chen, D., Guang, W. and French, J. (2003). Conception, early pregnancy loss, and time to clinical pregnancy: a population-based prospective study. *Fertil. Steril.* 79, 577-584. 10.1016/S0015-0282(02)04694-0 12620443

[DEV201507C185] Wang, W., Vilella, F., Alama, P., Moreno, I., Mignardi, M., Isakova, A., Pan, W., Simon, C. and Quake, S. R. (2020). Single-cell transcriptomic atlas of the human endometrium during the menstrual cycle. *Nat. Med.* 26, 1644-1653. 10.1038/s41591-020-1040-z 32929266

[DEV201507C186] Wang, F., Jia, W., Fan, M., Shao, X., Li, Z., Liu, Y., Ma, Y., Li, Y. X., Li, R., Tu, Q. et al. (2021). Single-cell immune landscape of human recurrent miscarriage. *Genomics Proteomics Bioinformatics* 19, 208-222. 10.1016/j.gpb.2020.11.002 33482359 PMC8602400

[DEV201507C187] Wasser, S. K. and Barash, D. P. (1983). Reproductive suppression among female mammals: implications for biomedicine and sexual selection theory. *Q. Rev. Biol.* 58, 513-538. 10.1086/413545 6686686

[DEV201507C188] Webster, A. and Schuh, M. (2017). Mechanisms of aneuploidy in human eggs. *Trends Cell Biol.* 27, 55-68. 10.1016/j.tcb.2016.09.002 27773484

[DEV201507C189] Weimar, C. H., Kavelaars, A., Brosens, J. J., Gellersen, B., De Vreeden-Elbertse, J. M., Heijnen, C. J. and Macklon, N. S. (2012). Endometrial stromal cells of women with recurrent miscarriage fail to discriminate between high- and low-quality human embryos. *PLoS One* 7, e41424. 10.1371/journal.pone.0041424 22848492 PMC3405140

[DEV201507C190] Weiss, S., Jaermann, T., Schmid, P., Staempfli, P., Boesiger, P., Niederer, P., Caduff, R. and Bajka, M. (2006). Three-dimensional fiber architecture of the nonpregnant human uterus determined ex vivo using magnetic resonance diffusion tensor imaging. *Anat. Rec. A Discov. Mol. Cell Evol. Biol.* 288, 84-90. 10.1002/ar.a.20274 16345078

[DEV201507C191] Wilcox, A. J., Baird, D. D. and Weinberg, C. R. (1999). Time of implantation of the conceptus and loss of pregnancy. *N. Engl. J. Med.* 340, 1796-1799. 10.1056/NEJM199906103402304 10362823

[DEV201507C192] Wildman, D. E., Chen, C., Erez, O., Grossman, L. I., Goodman, M. and Romero, R. (2006). Evolution of the mammalian placenta revealed by phylogenetic analysis. *Proc. Natl. Acad. Sci. USA* 103, 3203-3208. 10.1073/pnas.0511344103 16492730 PMC1413940

[DEV201507C193] Wimsatt, W. A. (1975). Some comparative aspects of implantation. *Biol. Reprod.* 12, 1-40. 10.1095/biolreprod12.1.1 806310

[DEV201507C194] Winterhager, E. and Kidder, G. M. (2015). Gap junction connexins in female reproductive organs: implications for women's reproductive health. *Hum. Reprod. Update* 21, 340-352. 10.1093/humupd/dmv007 25667189

[DEV201507C195] Yamaguchi, M., Nakaoka, H., Suda, K., Yoshihara, K., Ishiguro, T., Yachida, N., Saito, K., Ueda, H., Sugino, K., Mori, Y. et al. (2022). Spatiotemporal dynamics of clonal selection and diversification in normal endometrial epithelium. *Nat. Commun.* 13, 943. 10.1038/s41467-022-28568-2 35177608 PMC8854701

[DEV201507C196] Yang, M., Rito, T., Metzger, J., Naftaly, J., Soman, R., Hu, J., Albertini, D. F., Barad, D. H., Brivanlou, A. H. and Gleicher, N. (2021). Depletion of aneuploid cells in human embryos and gastruloids. *Nat. Cell Biol.* 23, 314-321. 10.1038/s41556-021-00660-7 33837289

[DEV201507C197] Yeaman, G. R., Guyre, P. M., Fanger, M. W., Collins, J. E., White, H. D., Rathbun, W., Orndorff, K. A., Gonzalez, J., Stern, J. E. and Wira, C. R. (1997). Unique CD8+ T cell-rich lymphoid aggregates in human uterine endometrium. *J. Leukoc. Biol.* 61, 427-435. 10.1002/jlb.61.4.427 9103229

[DEV201507C198] Yeaman, G. R., Collins, J. E., Fanger, M. W., Wira, C. R. and Lydyard, P. M. (2001). CD8+ T cells in human uterine endometrial lymphoid aggregates: evidence for accumulation of cells by trafficking. *Immunology* 102, 434-440. 10.1046/j.1365-2567.2001.01199.x 11328377 PMC1783206

[DEV201507C199] Yu, L., Ezashi, T., Wei, Y., Duan, J., Logsdon, D., Zhan, L., Nahar, A., Arteaga, C. A. P., Liu, L., Stobbe, C. et al. (2022). Large scale production of human blastoids amenable to modeling blastocyst development and maternal-fetal crosstalk. *bioRxiv* 10.1101/2022.09.14.507946 37683605

[DEV201507C200] Zilberberg, E., Smith, R., Nayot, D., Haas, J., Meriano, J., Barzilay, E. and Casper, R. F. (2020). Endometrial compaction before frozen euploid embryo transfer improves ongoing pregnancy rates. *Fertil. Steril.* 113, 990-995. 10.1016/j.fertnstert.2019.12.030 32386621

[DEV201507C201] Zinaman, M. J., Clegg, E. D., Brown, C. C., O'connor, J. and Selevan, S. G. (1996). Estimates of human fertility and pregnancy loss. *Fertil. Steril.* 65, 503-509. 10.1016/S0015-0282(16)58144-8 8774277

